# miR-22 Is a Novel Mediator of Vascular Smooth Muscle Cell Phenotypic Modulation and Neointima Formation

**DOI:** 10.1161/CIRCULATIONAHA.117.027799

**Published:** 2018-04-23

**Authors:** Feng Yang, Qishan Chen, Shiping He, Mei Yang, Eithne Margaret Maguire, Weiwei An, Tayyab Adeel Afzal, Le Anh Luong, Li Zhang, Qingzhong Xiao

**Affiliations:** 1 ^1^Department of Cardiology, The First Affiliated Hospital, School of Medicine, Zhejiang University, Hangzhou, China (F.Y., Q.C., M.Y., L.Z.).; 2 ^2^Centre for Clinical Pharmacology, William Harvey Research Institute, Barts and The London School of Medicine and Dentistry, Queen Mary University of London, United Kingdom (F.Y., S.H., E.M.M., W.A., T.A.A., L.A.L., Q.X.).; 3 ^3^Key Laboratory of Cardiovascular Diseases, The Second Affiliated Hospital, School of Basic Medical Sciences, Guangzhou Medical University, Xinzao Town, Panyu District, China (Q.X.).; 4 ^4^Key Laboratory of Protein Modification and Degradation, School of Basic Medical Sciences, Guangzhou Medical University, Xinzao Town, Panyu District, China (Q.X.).

**Keywords:** atherosclerosis, cell movement, cell proliferation, MDS1 and EVI1 complex locus protein, methyl-CpG-binding protein 2, microRNAs, neointima

## Abstract

**Background:**

MicroRNA-22 (miR-22) has recently been reported to play a regulatory role during vascular smooth muscle cell (VSMC) differentiation from stem cells, but little is known about its target genes and related pathways in mature VSMC phenotypic modulation or its clinical implication in neointima formation following vascular injury.

**Methods:**

We applied a wire-injury mouse model, and local delivery of AgomiR-22 or miR-22 inhibitor, as well, to explore the therapeutic potential of miR-22 in vascular diseases. Furthermore, normal and diseased human femoral arteries were harvested, and various in vivo, ex vivo, and in vitro models of VSMC phenotype switching were conducted to examine miR-22 expression during VSMC phenotype switching.

**Results:**

Expression of miR-22 was closely regulated during VSMC phenotypic modulation. miR-22 overexpression significantly increased expression of VSMC marker genes and inhibited VSMC proliferation and migration, whereas the opposite effect was observed when endogenous miR-22 was knocked down. As expected, 2 previously reported miR-22 target genes, MECP2 (methyl-CpG binding protein 2) and histone deacetylase 4, exhibited a regulatory role in VSMC phenotypic modulation. A transcriptional regulator and oncoprotein, EVI1 (ecotropic virus integration site 1 protein homolog), has been identified as a novel miR-22 target gene in VSMC phenotypic modulation. It is noteworthy that overexpression of miR-22 in the injured vessels significantly reduced the expression of its target genes, decreased VSMC proliferation, and inhibited neointima formation in wire-injured femoral arteries, whereas the opposite effect was observed with local application of a miR-22 inhibitor to injured arteries. We next examined the clinical relevance of miR-22 expression and its target genes in human femoral arteries. We found that miR-22 expression was significantly reduced, whereas MECP2 and EVI1 expression levels were dramatically increased, in diseased in comparison with healthy femoral human arteries. This inverse relationship between miR-22 and MECP2 and EVI1 was evident in both healthy and diseased human femoral arteries.

**Conclusions:**

Our data demonstrate that miR-22 and EVI1 are novel regulators of VSMC function, specifically during neointima hyperplasia, offering a novel therapeutic opportunity for treating vascular diseases.

## Abstract 2

Supplemental Digital Content is available in the text.

**Editorial, see p 1842**

**Clinical Perspective**

**What Is New?**

- We show that microRNA-22 (miR-22) is a novel mediator of vascular smooth muscle cell phenotypic modulation and neointima formation.
- We demonstrate that miR-22 controls vascular smooth muscle cell phenotype and injury-induced arterial remodeling by modulating multiple target genes (MECP2, HDAC4, and EVI1).
- We observe that miR-22 expression is supressed in the human femoral arteries with atherosclerotic plaques and have uncovered an inverse relationship between miR-22 and its target genes in healthy and diseased arteries.

**What Are the Clinical Implications?**

- Our findings on the miR-22–EVI1 and miR-22–MECP2 signaling axes in vascular smooth muscle cell phenotypic modulation present miR-22 and its target genes (EVI1 and MECP2) as novel biomarkers for peripheral arterial diseases.
- Local delivery of miR-22 in the injured arteries prevents adverse arterial remodeling, suggesting that the site-specific delivery of miR-22 mimics as a potential therapy for in-stent restenosis.

Vascular smooth muscle cell (VSMC) phenotype switching, or the phenotypic modulation of VSMCs from a differentiated, contractile state to a dedifferentiated, synthetic phenotype, has been shown to play a vital role in intima remodeling and in many cardiovascular diseases.^1^ Various environmental stimuli, such as growth factors, reactive oxidative species, and even mechanical injury, have been identified to lead to dramatic changes in VSMC phenotype and behavior.^1^ Both transcriptional and epigenetic mechanisms have been extensively implicated in VSMC phenotype switching and regulation of smooth muscle cell (SMC)–selective marker genes such as smooth muscle α-actin (SMαA), smooth muscle 22α (SM22α), smooth muscle calponin (CNN1), smooth muscle myosin heavy chain (SM-myh11), and smoothelin-B (SMTN-B).^1,2^ Of great interest to us is the growing evidence that supports a role for a novel class of gene regulators, microRNAs (miRs), in regulating VSMC differentiation from stem/progenitor cells and VSMC phenotype switching in response to vascular injury.^3–6^ Despite the growing number of identified miRs that have been implicated in VSMC differentiation and phenotypic modulation in response to injury, such as miR-1,^7^ miR-15b/16,^8^ miR-21,^9,10^ miR-34a,^11,12^ miR-133,^13^ miR-143/145 cluster,^14–20^ miR-182-3p,^21^ miR-214,^22^ miR-221/222,^23,24^ miR-638,^25^ and miR-663,^26^ the epigenetic regulation of VSMC phenotype switching has yet to be fully understood.

miR-22, originally proposed to act as a tumor suppressor,^27–29^ has been implicated in a variety of cardiac diseases^30–33^ and recently reported to play a regulatory role during VSMC differentiation from stem cells.^34^ However, little is known about its downstream targets and whether there is functional involvement of miR-22 in mature VSMC phenotypic modulation and vascular injury–induced neointima formation. In this study, we have identified EVI1 (ecotropic virus integration site 1 protein homolog) as a novel target of miR-22 and have demonstrated that the miR-22/EVI1 signaling axis plays an important role in VSMC phenotype switching and arterial remodeling in both mouse and human femoral artery disease models. Our work offers a possible mechanistic basis for the beneficial effect of EVI1 inhibition using arsenic trioxide–eluting stent (AES) on in-stent restenosis.

## Methods

The data that support the findings of this study are available from the corresponding author on reasonable request.

### miR-22 Promoter, EVI1 3′–Untranslated Region Reporter, and Mutation of miR-22 Binding Site Within EVI1 3′–Untranslated Region Reporter

miR-22 promoter DNA was amplified from mouse genomic DNA by polymerase chain reaction using primers shown in Table I in the online-only Data Supplement. Amplified DNA fragments were cloned into the Kpn I and Mlu I sites of the pGL3-basic vector (Promega), designated as pGL3-miR-22. Reporter vectors harboring 3′–untranslated region (3′-UTR) sequences of the murine EVI1 were created using cDNA from VSMCs. The 3′-flanking 3′-UTR (11-1142 nucleotides of the 3′-UTR region) of murine EVI1 gene (NM_007963) was amplified by polymerase chain reaction with primers shown in Table I in the online-only Data Supplement and cloned into the Sac I and Mlu I sites of the pmiR-reporter-basic vector (Ambion, Applied Biosystems), designated as pmiR-Luc-EVI1-WT. miR-22 binding site mutation was introduced into pmiR-Luc-EVI1 by using the QuikChange site-directed mutagenesis kit (Agilent Technologies) according to the manufacturer’s instructions and designated as pmiR-Luc-EVI1-mutant. All plasmids were verified by DNA sequencing at GATC Biotech.

### Mouse Femoral Artery Denudation Injury and Perivascular Delivery of miR-22 AgomiRs or LNA-miR-22

Mouse femoral arterial injury models were performed as described in our previous studies.^35–37^ To investigate the therapeutic effects of miR-22 on wire injury–induced vascular remodeling, 100 μL of 30% pluronic gel containing chemically modified and cholesterol-conjugated miR-22 (2.5 nmol) or scrambled AgomiRs were applied perivascularly to the femoral artery for local delivery of AgomiRs. miR-22 loss of function was conducted by local application of LNA-miR-22 (locked nucleic acid–modified miR-22 inhibitor) in the injured arteries. All animal experiments were conducted according to the Animals (Scientific Procedures) Act of 1986 (United Kingdom). All the animal procedures were approved by the Queen Mary University of London ethics review board (Project license No. 70/7216) and conform to the guidelines from Directive 2010/63/EU of the European Parliament on the protection of animals used for scientific purposes or the National Institutes of Health guidelines (Guide for the Care and Use of Laboratory Animals).

### Human Healthy and Diseased Femoral Arteries Collection and Immunohistochemistry Analysis

Human healthy and diseased femoral arterial specimens were collected from patients aged 30 to 80 years who underwent elective major lower extremity amputation of the index leg at the First Affiliated Hospital of Zhejiang University (China) between June 2013 and August 2017. Healthy femoral arterial specimens were obtained from patients with conditions unrelated to peripheral artery disease (eg, trauma). Diseased femoral arterial specimens were characterized by the presence of SMC-rich atherosclerotic lesions (identified by hematoxylin and eosin staining) and obtained from patients with peripheral artery disease. Individuals selected for healthy and diseased groups were age- and sex-matched. Exclusion criteria for enrollment were established ahead of time, including the presence of liver failure, dialysis because of renal failure, cancer, chemotherapy, and pregnancy, and the lack of consent to participate to the study, as well. Patient characteristics, including demographics, comorbidities, and medical treatments, were summarized in Table II in the online-only Data Supplement. All patients gave their written, informed consent to sample collection. All procedures had local ethical approval. All studies were approved by the Research Ethics Committees of the First Affiliated Hospital of Zhejiang University (institutional review board approval No. 2013/150), and all experiments were conducted according to the principles expressed in the Declaration of Helsinki.^38^ All human arterial specimens (1.5 cm per specimen) were divided into 2 portions: 1 portion (1.0 cm per specimen) was used to extract total RNA with miRCURY RNA Isolation Kits (formalin-fixed paraffin-embedded) (EXIQON, 300115), and the other portion (0.5 cm per specimen) underwent fixing with 4% formaldehyde for hematoxylin and eosin staining, as detailed previously.^39^ All the human arterial specimens were examined by 2 independent cardiovascular pathologists.

### Statistical Analysis

Statistical analysis was performed by using Graphpad Prism5. Numbers (n) refer to the number of independent experiments, mice, or patients. The Shapiro-Wilk normality test was used for checking the normality of the data, where data with a Shapiro-Wilk test *P* value of >0.05 were considered to fit a normal distribution. A two-tailed unpaired Student *t* test and 1- (or 2-) way ANOVA with Dunnett (or Bonferroni) post hoc test were applied for comparisons between 2 or multiple groups, respectively, if the data displayed a normal distribution. Conversely, a nonparametric Mann-Whitney *U* test was applied for comparing 2 groups if the data did not display normal distribution. Spearman rank correlation analyses were conducted to characterize the relationships between the gene expression levels of miR-22 and its target genes, MECP2 (methyl-CpG binding protein 2) and EVI1. The Fisher exact test was used to compare the significance for categorical variables (such as patient demographics, comorbidities, hospital characteristics) between patients with healthy and diseased femoral arteries. *P* < 0.05 was considered as statistically significant.

Additional detailed description of materials and methods is provided in the online-only Data Supplement.

## Results

### miR-22 Expression Is Modulated During VSMC Phenotype Switching In Vivo, Ex Vivo, and In Vitro

**Figure 1.**
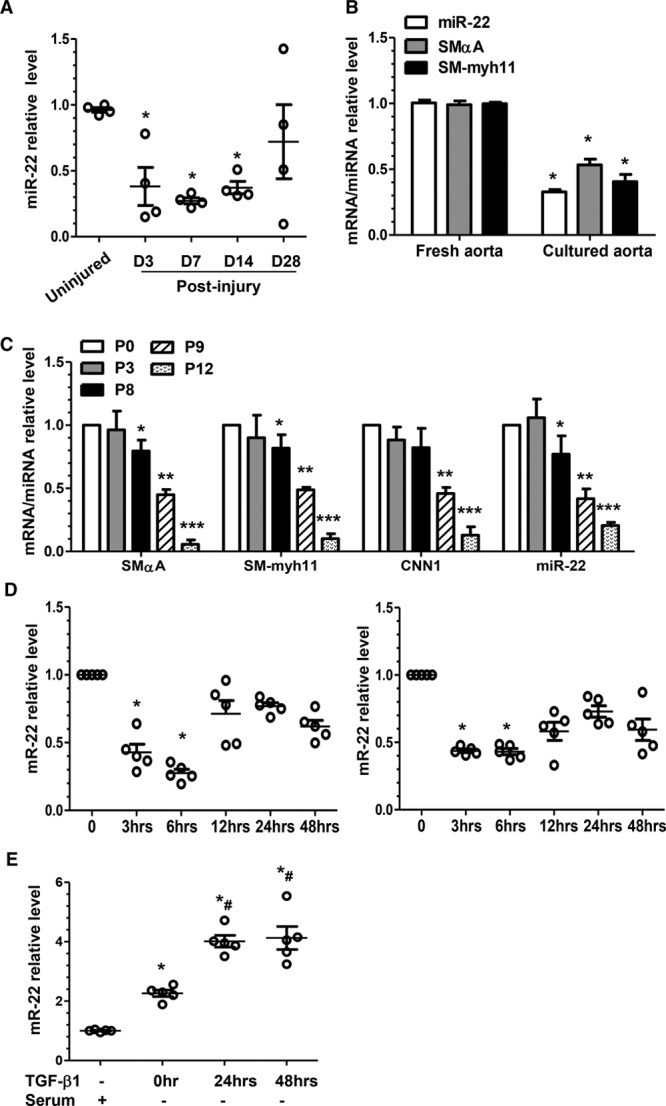
**miR-22 expression is closely modulated during VSMC phenotype switching. A**, miR-22 was significantly downregulated after injury in the in vivo mouse model. Total RNA was collected from the uninjured (day [D] 0) and injured femoral arteries (D3, D7, D14, and D28 postinjury). RT-qPCR analyses were conducted to obtain relative expression levels. **B**, miR-22 was decreased in the ex vivo cultured thoracic aortas. RT-qPCR analysis was used to examine the mRNA (SMαA and SM-myh11) and miR (miR-22) levels in the freshly isolated aortas and the aortas cultured in DMEM containing 20% serum for 3 days. Data and error bars in **A** and **B** represent the mean±SEM (n=4), where up to 5 femoral arteries were pooled as 1 experiment. *P<0.05 (versus D0 [**A**], or fresh aorta [**B**]). **C**, miR-22 expression was downregulated in the extended culture of murine VSMCs. Total RNA, including miR (miR-22) and mRNA (SMαA, SM-myh11, and CNN1), was harvested from freshly cultured VSMCs (cultured until day 7 and then split, designated P0), and VSMCs with the indicated passage number (P0, P3, P8, P9, or P12) were subjected to RT-qPCR analysis to obtain relative expression levels. **D**, Serum (**left**) and PDGF-BB (**right**) downregulated miR-22 in cultured VSMCs. VSMCs were subjected to serum starvation for 48 hours, followed by incubation with 20% serum or PDGF-BB (10 ng/mL), respectively. Total RNA was harvested at each indicated time point. **E**, Serum starvation and TGF-β1 upregulated miR-22 in cultured VSMCs. VSMCs in normal culture were used as control (TGF-β1–, Serum+) or subjected to serum starvation for 48 hours and then harvested after the indicated stimulations and time points: no stimulation (TGF-β1–, Serum–) at 0 hour, TGF-β1 stimulation at 24 hours (TGF-β1-24hrs, Serum–), and TGF-β1 stimulation at 48 hours (TGF-β1-48hrs, Serum–). Total RNA was extracted and subjected to RT-qPCR analysis with a specific miR-22 forward primer and a universal miR reverse primer. Data and error bars in **C** through **E** represent mean±SEM (n=5). *P<0.05, **P<0.01, ***P<0.001 (versus P0 [**C**], 0 hours [**D**], or normal culture [**E**]); #*P*<0.05 (versus 0 hours). DMEM indicates Dulbecco’s modified Eagle’s medium; miR-22, microRNA-22; PDGF-BB, platelet-derived growth factor BB; RT-qPCR, reverse transcription quantitative polymerase chain reaction; TGF-β1, transforming growth factor β1; and VSMC, vascular smooth muscle cell.

To explore the potential function of miR-22 in VSMC phenotype switching, we examined in vivo, ex vivo, and in vitro mouse models of VSMC phenotype switching. Reverse transcription quantitative polymerase chain reaction (RT-qPCR) data showed that miR-22 was significantly decreased in the injured versus uninjured femoral arteries (Figure 1A, in vivo). RT-qPCR data also showed downregulation of miR-22, and the VSMC markers (SmαA and SM-myh11), as well, in the explanted cultured thoracic aortic tissues (Figure 1B, ex vivo). As expected, we found that gene expression levels of VSMC markers were maintained in the VSMCs in early passages (up to passage 8, P8) but significantly downregulated later (P9 to P12) (Figure 1C, in vitro). Thus, VSMCs between P5 and P8 were used in the current study. It is interesting to note that a similar decreased expression pattern was observed for miR-22 (Figure 1C). Furthermore, miR-22 expression in cultured VSMCs was reduced in response to platelet-derived growth factor BB (PDGF-BB) and serum stimulation (Figure 1D), whereas the opposite effect was seen in the serum-starved VSMCs (Figure 1E). The induction of miR-22 in VSMCs was further enhanced by transforming growth factor β1 (TGF-β1) treatment 24 hours and 48 hours after serum starvation (Figure 1E). Altogether, these data confirm that expression of miR-22 is altered during phenotype switching in vivo, ex vivo, and in vitro.

### miR-22 Is Transcriptionally Regulated During VSMC Phenotypic Modulation

**Figure 2.**
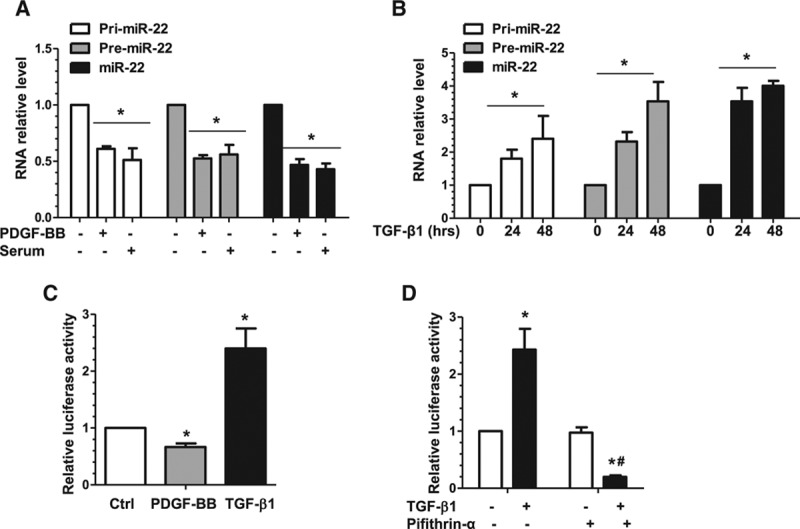
**miR-22 is transcriptionally regulated in VSMC phenotypic modulation. A**, PDGF-BB and serum significantly downregulated miR-22, and its primary (pri-miR-22) and precursor (premiR-22) transcripts, as well. Serum-starved VSMCs were used as the control (PDGF-BB–, Serum–), incubated with serum (20%), or incubated with PDGF-BB (10 ng/mL) for 3 hours. Total RNA was harvested and subjected to RT-qPCR analysis to examine miR-22 and its transcripts. **B**, TGF-β1 significantly upregulated miR-22, pri-miR-22, and premiR-22. RT-qPCR analysis was used to examine the miR levels in serum-starved VSMCs incubated with TGF-β1 (5 ng/mL) for 0, 24, and 48 hours. **C**, PDGF-BB significantly decreases, whereas TGF-β1 significantly increases miR-22 gene promoter activity. VSMCs transfected with miR-22 gene promoter (pGL3-miR-22) were subjected to serum starvation for 24 hours, followed by mock treatment (Ctrl) or incubation with PDGF-BB (10 ng/mL) or TGF-β1 (5 ng/mL) for 6 hours. Cell lysates were harvested and analyzed using the luciferase activity assay. **D**, TGF-β1 upregulated miR-22 in a P53-depedent manner. VSMCs transfected with miR-22 gene promoter (pGL3-miR-22) were subjected to serum starvation for 24 hours, followed by incubation with vehicle (TGF-β1–, Pifithrin-α–) or TGF-β1 (5 ng/mL) for 24 hours in the absence or presence of 15 µmol/L P53-specific inhibitor, Pifithrin-α. Cell lysates were harvested and subjected to the luciferase activity assay. Data and error bars in **A** through **D** represent the mean±SEM (n=3 in **A**, **C**, and **D**; n=4 in **B**). *P<0.05 (versus PDGF-BB–Serum– [**A**], 0 hours [**B**], Ctrl [**C**], or TGF-β1– [**D**]); ^#^*P*<0.05 (versus TGF-β1+, Pifithrin-α–). miR-22 indicates microRNA-22; PDGF-BB, platelet-derived growth factor BB; RT-qPCR, reverse transcription quantitative polymerase chain reaction; TGF-β1, transforming growth factor β1; and VSMC, vascular smooth muscle cell.

Transcriptional modulation and regulation of microRNA biogenesis are 2 main mechanisms through which miR activity can be regulated. Here, we sought to determine whether either of these mechanisms was responsible for regulating miR-22 expression during VSMC phenotype switching in cultured VSMCs. We measured expression of miR-22, its primary (Pri-miR-22) transcript, and its precursor (Pre-miR-22) transcript. We found that all were significantly downregulated by PDGF-BB and serum (Figure 2A) but upregulated by TGF-β1 (Figure 2B), suggesting that miR-22 was transcriptionally regulated during VSMC phenotypic modulation. Such a notion was further supported by data from our luciferase activity assays using miR-22 gene promoter reporter (Figure 2C). A recent study showed that miR-22 expression is regulated by a P53-dependent mechanism during cardiac aging,^30^ and such a mechanism may be responsible for miR-22 regulation during VSMC phenotypic modulation. Indeed, we found that the P53-specific inhibitor, Pifithrin-α (15 µmol/L), actively reduced miR-22 expression in TGF-β1–treated VSMCs (Figure 2D). These data demonstrate that TGF-β1 can regulate miR-22 transcription in VSMCs, likely through a P53-dependent mechanism.

### Functional Role of miR-22 in VSMC Phenotype Switching

**Figure 3.**
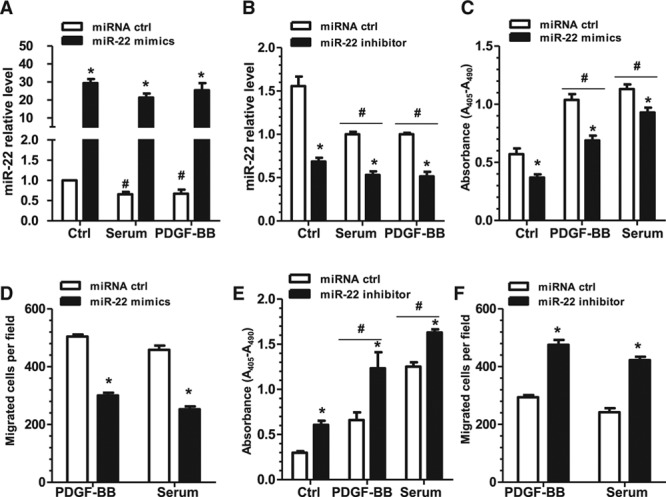
**miR-22 modulates VSMC proliferation and migration.** VSMCs were transfected with miR-22 mimics, miR-22 inhibitor, or respective negative control miR (miRNA ctrl), followed by 24 hours of serum starvation. Subsequently, cells were treated with vehicle control (Ctrl), PDGF-BB (10 ng/mL), or serum (20%) for 48 hours and analyzed. **A**, RT-qPCR analysis confirmed miR-22 overexpression in VSMCs transfected with miR-22 mimics on PDGF-BB and serum treatment. **B**, RT-qPCR analysis confirmed that miR-22 expression was significantly inhibited in VSMCs transfected with miR-22 inhibitor in all treatments. **C**, BrdU assays revealed significantly decreased absorbance of VSMCs transfected with miR-22 mimics in all treatments, indicating decreased proliferation. **D**, Transwell migration assays showed significantly decreased migration of VSMCs transfected with miR-22 mimics under both PDGF-BB and serum stimulation. **E**, BrdU assays revealed significantly increased absorbance and therefore proliferation of VSMCs transfected with the miR-22 inhibitor on PDGF-BB and serum treatment. **F**, Transwell migration assays showed significantly increased migration of VSMCs transfected with the miR-22 inhibitor under both PDGF-BB and serum stimulation. Note: No or very few migrated cells were observed without cell chemoattractant in transwell migration assays; therefore, no control treatment is shown. Data and error bars represent the mean±SEM (n=3 in **A**; 4 in **B**, **C**, and **E**; or 5 in **D** and **F**). *P<0.05 (versus miRNA ctrl). ^#^*P*<0.05 (versus Ctrl). BrdU indicates bromodeoxyuridine; miR-22, microRNA-22; PDGF-BB, platelet-derived growth factor BB; RT-qPCR, reverse transcription quantitative polymerase chain reaction; and VSMC, vascular smooth muscle cell.

To test our hypothesis that miR-22 plays a role in VSMC phenotype switching, we transfected murine and human VSMCs with miR-22 mimics or miR-22 inhibitor and then subjected the transfected cells to various analyses. RT-qPCR data showed that miR-22 expression in murine VSMCs was significantly upregulated by miR-22 mimics (Figure IA in the online-only Data Supplement) and downregulated by the miR-22 inhibitor (Figure IB in the online-only Data Supplement). The expression of 4 VSMC genes (SMαA, SM22α, SM-myh11, and SMTN-B) was also significantly increased in VSMCs transfected with miR-22 mimics (Figure IA in the online-only Data Supplement) and significantly decreased in VSMCs treated with the miR-22 inhibitor (Figure IB in the online-only Data Supplement). It is important to note that we found that, whereas PDGF-BB treatment reduced expression of SM-myh11 and SMTN-B, the addition of miR-22 mimics significantly increased expression of both genes even with PDGF-BB treatment (Figure IC and IDin the online-only DataSupplement), suggesting that miR-22–induced contractile gene expression is PDGF-BB–independent. RT-qPCR analysis verified that miR-22 was successfully overexpressed and knocked down in VSMCs by miR-22 mimics and inhibitor, respectively, in control, serum, and PDGF-BB treatments (Figure 3A and 3B). It is important to note that VSMC proliferation, growth, and migration were significantly inhibited by miR-22 overexpression as demonstrated in bromodeoxyuridine incorporation analysis (Figure 3C), cell counting (Figure IIA in the online-only Data Supplement), transwell migration (Figure 3D), and wound-healing assays (Figure IIB in the online-only Data Supplement). Conversely, an increased capacity to proliferate, grow, and migrate was observed in VSMCs transfected with the miR-22 inhibitor (Figure 3E and 3F, Figure IIC and IID in the online-only Data Supplement). Similar findings from human aortic SMCs also supported that miR-22 plays an important role during human VSMC phenotypic modulation (Figure III in the online-only Data Supplement).

### VSMC Apoptosis Is Not Regulated by miR-22

Apart from VSMC migration and proliferation, VSMC apoptosis has been suggested to be another major contributing factor to atherosclerotic lesion formation and plaque phenotype.^40–42^ To explore any potential roles of miR-22 in VSMC apoptosis, serum-starved VSMCs were subjected to extended serum starvation (96 hours) (Figure IVA and IVB in the online-only Data Supplement) or incubation with 10 µmol/L H_2_O_2_ (Figure IVC and IVD in the online-only Data Supplement) to induce apoptosis. Data from flow cytometry analyses showed that the percentages of live, early apoptotic, late apoptotic, and necrotic VSMCs were not significantly changed by either miR-22 overexpression (Figure IVA and IVC in the online-only Data Supplement) or inhibition (Figure IVB and IVD in the online-only Data Supplement), indicating that miR-22 was not involved in either extended serum starvation or H_2_O_2_-induced VSMC apoptosis.

### Potential Downstream Targets of miR-22 During VSMC Phenotype Switching

Conserved miR-22 binding site(s) were found within 270 genes by using Targetscan. Among them, 23 validated/predicted targets of miR-22 were selected for further study, because they are known to play key regulatory roles in both VSMC functions (differentiation, proliferation, migration, apoptosis, and cell cycle)^11,43–45^ and vascular biology^34^ (Figure V in the online-only Data Supplement). Our data showed that the expression levels of TP53INP1, TGFRβ1, P21, MAP2K4, and SP1 were significantly increased in both serum-starved and TGF-β1–treated VSMCs, whereas the expression levels of MECP2, ARPC5, EVI1, MYST4, and histone deacetylase 4 (HDAC4) were significantly inhibited by either serum starvation or TGF-β1 stimulation (Figure V in the online-only Data Supplement). It is important to note that MECP2 and EVI1 expression in serum-starved VSMCs was further decreased by TGF-β1 treatment (Figure V in the online-only Data Supplement). Because TGF-β1 treatment increased miR-22 expression (Figure 1E) and decreased expression of MECP2, EVI1, and HDAC4, these observations indicated these genes could be potential targets of miR-22.

### MECP2 and HDAC4 Are 2 Functional Target Genes of miR-22s During VSMC Phenotype Switching

Our previous study showed that miR-22 targets MECP2 during VSMC differentiation from stem cells,^34^ leading to our hypothesis that MECP2 may also be a downstream target of miR-22 during VSMC phenotype switching. Data from RT-qPCR and Western blot analyses showed that both MECP2 mRNA and protein expression levels are decreased in VSMCs by miR-22 overexpression (Figure VIA and VIB in the online-only Data Supplement). The MECP2 3′-UTR reporter^34^ activity was also significantly inhibited by miR-22 mimics (Figure VIC in the online-only Data Supplement). Moreover, all 5 VSMC genes (SMαA, SM22α, CNN1, SM-myh11, SMTN-B), but not miR-22, were significantly upregulated by MECP2 inhibition (Figure VID in the online-only Data Supplement), supporting that MECP2 is the downstream target of miR-22. It is important to note that VSMC proliferation (Figure VIE in the online-only Data Supplement) and migration (Figure VIF in the online-only Data Supplement) were significantly decreased by MECP2 knockdown, demonstrating that MECP2 suppression can recapitulate the effects of miR-22 overexpression in VSMC phenotypic modulation. In addition to MECP2, HDAC4 is another reported target of miR-22.^33,46^ miR-22 overexpression decreased the expression level of HDAC4 (Figure VIIA in the online-only Data Supplement). Inhibition of HDAC4 mimics miR-22 overexpression during VSMC phenotype switching (Figure VIIB through VIID in the online-only Data Supplement). These data suggested that miR-22 also targets HDAC4 in mature VSMCs.

### EVI1 Is a Novel Target Gene and Responsible for miR-22–Mediated VSMC Phenotype Switching

**Figure 4.**
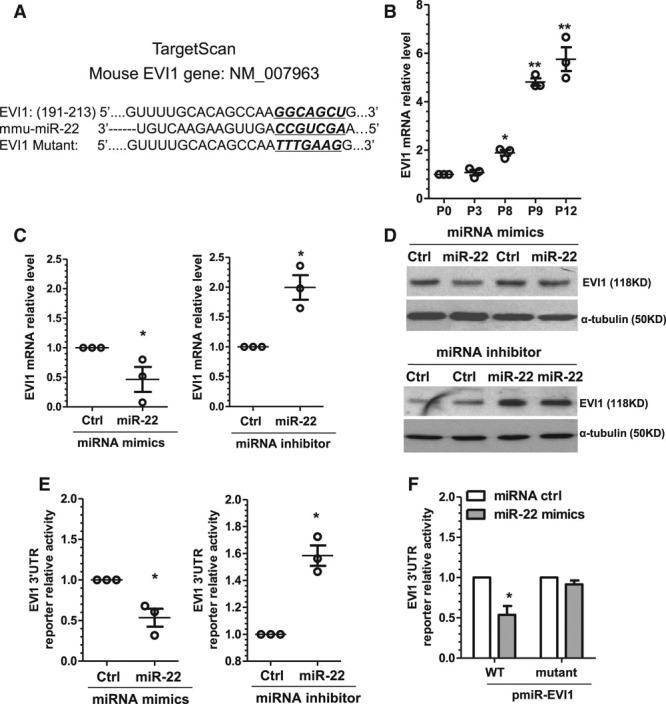
**EVI1 is the novel target of miR-22 in VSMCs. A**, The predicted miR-22 binding site within EVI1 3′-UTR by Targetscan. EVI1, miR-22 sequence (mmu-miR-22), and the miR-22 binding site mutant (EVI1 mutant) are depicted in this illustration. The mutation site in EVI1 mutant and corresponding sequences in wild-type EVI1 and mmu-miR-22 are underlined and bold. **B**, EVI1 was upregulated in the extended cultured VSMCs. Each dot represents EVI1 mRNA level in each passage (P3, P8, P9, P12) normalized to EVI1 mRNA level of P0. **C** and **D**, EVI1 was negatively regulated by miR-22. VSMCs transfected with miR-22 mimics, inhibitor, or respective controls (Ctrl), as indicated, were subjected to serum starvation for 48 hours. Total RNA and protein were harvested and subjected to RT-qPCR (**C**) and Western blot (**D**) analyses, respectively. **E**, miR-22 repressed EVI1 3′-UTR reporter activity. miR-22 mimics, inhibitor, or respective controls (Ctrl) were cotransfected with EVI1 3′-UTR reporter into VSMCs, as indicated. Transfected cells were subjected to serum starvation for 48 hours, and cell lysates were subjected to luciferase activity assay. **F**, miR-22 binding site was required for miR-22–mediated EVI1 gene repression. miR-22 mimics or control miR mimics (miRNA ctrl) were cotransfected into VSMCs with wild-type reporter (pmiR-EVI1-WT) or the reporter containing mutated miR-22 binding site (pmiR-EVI1-mutant). Transfected cells were subjected to serum starvation for 48 hours before luciferase activity assay. Data and error bars in **B** through **F** are representative (**D**) or mean±SEM (**B**, **C**, **E**, and **F**) (n=4 in **D** and **F**). *P<0.05, **P<0.01 (versus P0 or respective miRNA ctrl). EVI1 indicates ecotropic virus integration site 1 protein homolog; miR-22, microRNA-22; RT-qPCR, reverse transcription quantitative polymerase chain reaction; 3′-UTR, 3′-untranslated region; and VSMC, vascular smooth muscle cell.

The transcriptional regulator and oncoprotein EVI1 was predicted as one of the target genes of miR-22 by Targetscan (Figure 4A). Opposite from miR-22 (Figure 1C), EVI1 gene expression was dramatically increased in the extended passages (P8, P9, and P12) of cultured murine VSMCs (Figure 4B). These observations prompted us to investigate whether or not EVI1 is a novel target gene of miR-22 during VSMC phenotype switching. As expected, expression levels of both the EVI1 mRNA (Figure 4C) and protein (Figure 4D) were significantly downregulated by miR-22 mimics but upregulated by miR-22 inhibitor in VSMCs. The luciferase activity of EVI1 3′-UTR reporter was significantly repressed by miR-22 mimics but enhanced by miR-22 inhibition (Figure 4E). Once the miR-22 binding site within EVI1 3′-UTR was mutated, the miR-22 mimic–induced inhibition of EVI1 expression was abrogated (Figure 4F), suggesting that miR-22 directly downregulates EVI1 via its 3′-UTR binding site.

**Figure 5.**
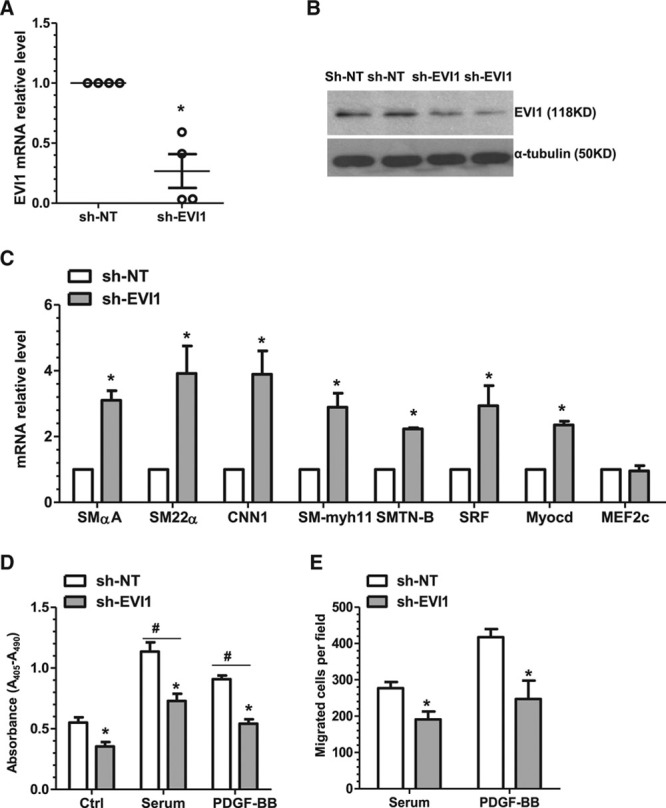
**EVI1 inhibition reproduces the effects of miR-22 overexpression on VSMC-specific gene expression, proliferation, and migration. A** and **B**, EVI1 knockdown VSMC was generated and validated. Total RNA and protein of control (nontarget shRNA, sh-NT) and EVI1 stable knockdown (EVI1 shRNA, sh-EVI1) VSMCs were harvested and subjected to RT-qPCR (**A**) and Western blot (**B**) analyses, respectively. **C**, EVI1 knockdown significantly increases expression of VSMC markers (SMαA, SM22α, CNN1, SM-myh11, SMTN-B) and transcription factors (SRF and Myocd), although the transcription factor MEF2c exhibited no significant change in expression. Total RNA of control and EVI1 stable knockdown VSMCs were harvested and subjected to RT-qPCR. **D**, Inhibition of endogenous EVI1 decreases VSMC proliferation. Control and EVI1 stable knockdown VSMCs were subjected to serum starvation for 48 hours, followed by BrdU incorporation assays in response to no (Ctrl), serum (20%), and PDGF-BB (10 ng/mL) stimulation. **E**, Inhibition of endogenous EVI1 decreases VSMC migration. Control and EVI1 stable knockdown VSMCs were subjected to serum starvation for 48 hours, followed by transwell migration in response to serum (20%) and PDGF-BB (30 ng/mL) stimulation. Note: No or very few migrated cells were observed without cell chemoattractant in transwell migration assays; therefore, no control treatment is shown. Data and error bars are representative (**B**) or mean±SEM (**A** and **C** through **E**) (n=3 in **C**; 4 in **D**; or 5 in **E**). *P<0.05 (versus sh-NT). ^#^*P*<0.05 (versus Ctrl). BrdU indicates bromodeoxyuridine; EVI1, ecotropic virus integration site 1 protein homolog; miR-22, microRNA-22; PDGF-BB, platelet-derived growth factor BB; RT-qPCR, reverse transcription quantitative polymerase chain reaction; shRNA, small hairpin RNA; and VSMC, vascular smooth muscle cell.

To explore the functional significance of EVI1 in VSMC phenotype switching, an EVI1 small hairpin RNA lentivirus was produced and used to generate a stable knockdown of EVI1 in VSMCs as validated by RT-qPCR (Figure 5A) and Western blot (Figure 5B). We found that expression of 5 VSMC genes and 2 SMC transcription factor genes (SRF and Myocd) was significantly increased in EVI1 knockdown VSMCs (Figure 5C). Similarly, VSMC proliferation (Figure 5D) and migration (Figure 5E) in response to both serum and PDGF-BB stimulation were significantly decreased. Altogether, our data suggest that, similar to overexpression of miR-22, EVI1 suppression in VSMCs simulates the effects of miR-22 overexpression on VSMC gene expression, proliferation, and migration.

To further understand the functional significance of EVI1 in miR-22–induced VSMC phenotype switching, control VSMCs and EVI1 knockdown VSMCs were transfected with either a control inhibitor or a miR-22 inhibitor. The gene expression data showed that both miR-22 and EVI1 were successfully downregulated in VSMCs by miR-22 inhibitor and EVI1 small hairpin RNA, respectively (Figure VIIIA in the online-only Data Supplement). The expression level of EVI1 was significantly increased by miR-22 inhibitor in control VSMCs, whereas the same inhibitory effect was abolished in EVI1 knockdown VSMCs (Figure VIIIA in the online-only Data Supplement). It is important to note that RT-qPCR data showed that miR-22 inhibition downregulated VSMC gene expression, and this inhibitory effect of miR-22 was abolished by EVI1 knockdown (Figure VIIIA in the online-only Data Supplement). Similar phenomena were observed in serum and PDGF-BB–induced VSMC proliferation (Figure VIIIB in the online-only Data Supplement) and migration (Figure VIIIC in the online-only Data Supplement). Taken together, we demonstrate that EVI1 is a novel target gene that is at least partially responsible for miR-22-mediated VSMC phenotype switching.

### EVI1 Is a Transcriptional Repressor for VSMC Marker Gene Expression

**Figure 6.**
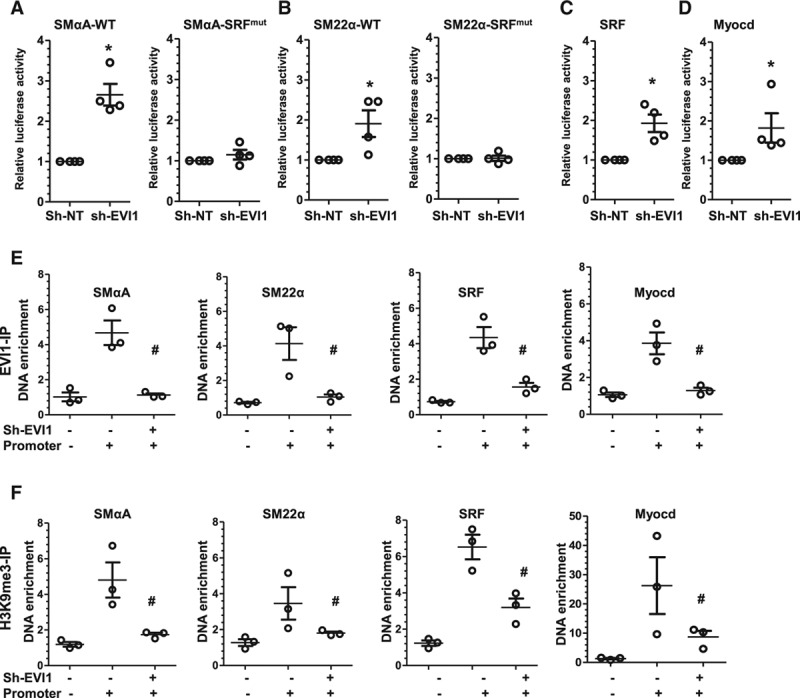
**EVI1 functions as a transcriptional repressor for VSMC gene expression. A** and **B**, SRF binding element(s) are required for EVI1-mediated SMαA and SM22α gene promoter activity. Wild-type (SMαA/SM22α) or SRF binding site(s) mutants (SRF^mut^) of SMαA (**A**) and SM22α (**B**) gene promoter reporters were transfected into control (nontarget shRNA, sh-NT) and EVI1 stable knockdown (EVI1 shRNA, sh-EVI1) VSMCs. Transfected cells were subjected to serum starvation for 48 hours, and cell lysates were subjected to luciferase activity assay. **C** and **D**, EVI1 inhibition significantly increases SRF and Myocd gene promoter activity. SRF (**C**) and Myocd (**D**) gene promoter reporters were transfected into control and EVI1 stable knockdown VSMCs. Transfected cells were subjected to serum starvation for 48 hours, and cell lysates were subjected to luciferase activity assay. **E**, EVI1 was significantly enriched at the promoter regions of SMαA (**far left**), SM22α (**middle left**), SRF (**middle right**), and Myocd (**far right**) genes and was significantly decreased by EVI1 knockdown, indicating direct binding of EVI1 to these promoter regions. ChIP assays were performed to measure EVI1 enrichment in the promoter region of its downstream targets. Control (sh-EVI1–) and EVI1 stable knockdown (sh-EVI1+) VSMCs were lysed and incubated with antibody against EVI1 to immunoprecipitate EVI1-bound promoter DNA, followed by qPCR to quantify DNA enrichment. For SMαA and SM22α, genomic DNA without SRF binding sites were amplified as additional control for specific promoter DNA enrichment and designated as Promoter–. For SRF and Myocd, PCR amplification of DNA region adjacent to their promoter was designated as Promoter–. **F**, H3K9me3 enrichment within the promoter regions of SMαA (**far left**), SM22α (**middle left**), SRF (**middle right**), and Myocd (**far right**) genes was significantly decreased by EVI1 knockdown. ChIP assays were performed by using an antibody against H3K9me3. Data and error bars are mean±SEM. *P<0.05 (versus sh-NT). ^#^*P*<0.05 (versus sh-EVI1–, Promoter+). ChIP indicates chromatin immunoprecipitation; EVI1, ecotropic virus integration site 1 protein homolog; qPCR, quantitative polymerase chain reaction; shRNA, small hairpin RNA; and VSMC, vascular smooth muscle cell.

As described above, gene expression data indicate that EVI1 plays an inhibitory role in regulating the expression of 5 VSMC genes and 2 transcription factors. Luciferase activity data showed that EVI1 knockdown induced both SMαA and SM22α gene promoter activity, but this was completely lost once the SRF binding element within the gene promoter was mutated (Figure 6A and 6B), confirming that EVI1 regulates VSMC marker expression through a transcriptional mechanism requiring SRF binding. Moreover, we found that both SRF and Myocd gene promoter reporter activity was significantly increased in EVI1 knockdown VSMCs (Figure 6C and 6D), indicating that EVI1 also represses SRF and Myocd gene regulation.

To understand the underlying molecular mechanisms of EVI1 regulation, chromatin immunoprecipitation assays were conducted in control and EVI1 knockdown VSMCs using an EVI1-specific antibody. We found significant enrichment of EVI1 within the promoter regions of SMαA, SM22α, SRF, and Myocd genes in control VSMCs (Figure 6E). Such enrichment disappeared in EVI1 knockdown VSMCs (Figure 6E), confirming that EVI1 directly binds to these 4 gene promoters. In our previous publication, H3K9me3 was shown to bind to promoter regions of VSMC marker genes and suppress their expression.^34^ This led to the hypothesis that H3K9me3-induced suppression is dependent on EVI1. A chromatin immunoprecipitation assay with a H3K9me3-specific antibody showed a significantly lower H3K9me3 enrichment within the promoter regions of VSMC marker genes in EVI1 knockdown cells (Figure 6F), suggesting that the binding of H3K9me3 to the promoter regions of VSMC genes is EVI1-dependent.

### Therapeutic Potential of miR-22 for Postinjury Arterial Remodeling

**Figure 7.**
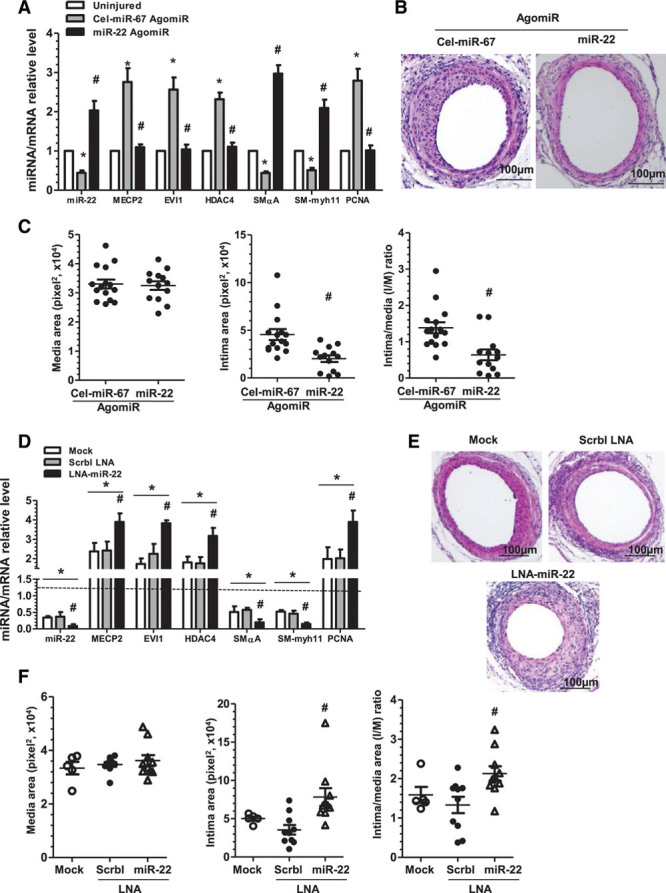
**Modulation of miR-22 expression in the injured arteries influences neointima formation. A** through **C**, Local enforced expression of miR-22 reduces neointima formation in the injured femoral arteries. After wire-induced injury, 100 µL of 30% pluronic gel containing 2.5 nmol control AgomiR (Cel-miR-67 AgomiR) or miR-22 AgomiR per artery per mouse was immediately applied and packed around the injured femoral arteries. Three days (**A**) or 4 weeks (**B** and **C**) later, injured segments of femoral arteries were harvested for analyses. **A**, Perivascular delivery of miR-22 AgomiRs reversed the gene expression profiles in wire-induced femoral artery injury. Total RNA was harvested from uninjured and AgomiR-applied injured femoral arteries before undergoing RT-qPCR analyses. Data and error bars represent mean±SEM (n=3) (5 femoral arteries were pooled for each experiment). *P<0.05 (versus uninjured arteries). #*P*<0.05 (miR-22 AgomiRs versus Cel-miR-67 AgomiRs in the injured arteries). **B** and **C**, Locally enforced expression of miR-22 inhibited neointima formation in wire-injured femoral arteries. Paraffin sections from both groups (n=15 mice for Cel-miR-67 and n=13 mice for miR-22 AgomiRs) were prepared and subjected to H&E staining. Representative images (**B**) and morphological characteristics (**C**), including media area (**left**), intima area (**middle**), and intima/media (I/M) ratio (**right**) at 4 weeks after injury were presented. #*P*<0.05 (versus Cel-miR-67 AgomiRs). **D** through **F**, miR-22 inhibition promotes neointima formation in the injured arteries. After wire injury, 100 µL of 30% pluronic gel containing vehicle (mock transfection, Mock), 2.5 nmol control LNA (scrambled LNA, Scrbl-LNA), or LNA-miR-22 per artery per mouse was immediately applied and packed around injured femoral arteries. Seven days (**D**) or 4 weeks (**E** and **F**) later, injured segments of femoral arteries were harvested for RT-qPCR analyses (**D**), H&E staining (**E**), and morphological quantification (**F**) (n=5 mice for Mock and n=10 mice for LNA-miR-22 and Scrbl-LNA). Note: Dotted line (**D**) represents the gene expression level in the uninjured arteries, which is set as 1.0. *P<0.05 (versus uninjured arteries). #*P*<0.05 (LNA-miR-22 versus Mock and Scrbl-LNA). EVI1 indicates ecotropic virus integration site 1 protein homolog; HDAC4, histone deacetylase 4; H&E, hematoxylin and eosin; LNA, locked nucleic acid; MECP2, methyl-CpG binding protein 2; miR-22, microRNA-22; PCNA, proliferating cell nuclear antigen; and RT-qPCR, reverse transcription quantitative polymerase chain reaction.

To determine the therapeutic potential of miR-22 in postinjury arterial remodeling, 2.5 nmol of miR-22 or Cel-miR-67 AgomiRs (negative control) was perivascularly applied to femoral arteries immediately after wire-induced injury as described in our previous studies.^12,22^ In comparison with uninjured arteries, injured arteries treated with control Cel-miR-67 AgomiRs displayed significantly decreased expression of miR-22 and VSMC genes (SMαA and SM-myh11) and significantly increased expression of cell proliferation marker gene, PCNA, and the identified miR-22 target genes (MECP2, HDAC4, and EVI1), whereas the opposite effects were observed in injured arteries treated with miR-22 AgomiR (Figure 7A). It is important to note that increased miR-22 expression via perivascular transfection of miR-22 AgomiR was specific to injured arteries, yet absent in other organs/tissues (eg, heart, skeletal muscle, spleen, liver, kidney, and lung) (data not shown). As expected, injury-induced MECP2, EVI1, HDAC4, and PCNA gene expression was blunted, whereas the expressions of VSMC genes (SMαA and SM-myh11) were enhanced by local ectopic expression of miR-22 (Figure 7A). These findings are consistent with the notion that miR-22 promoted VSMC phenotype switching from its proliferative, synthetic state to a contractile phenotype after vascular injury. Consequently, although the application of control AgomiRs (Cel-miR-67 AgomiR) in the injured artery exhibited pronounced neointima hyperplasia after 28 days, miR-22 overexpression significantly inhibited neointima formation, as evidenced by decreased intima area and intima/media ratio in the miR-22 AgomiR–treated injured artery, although the media area experienced no significant change (Figure 7B and 7C). To better understand the functional role of miR-22 in postinjury arterial remodeling, we also conducted miR-22 loss-of-function experiments using LNA-miR-22 and found that miR-22 inhibition produces the opposite effects of miR-22 overexpression on VSMC marker expression and injury-induced neointima hyperplasia (Figure 7D through 7F). These findings suggest therapeutic potential for harnessing local ectopic expression of miR-22 to suppress neointima hyperplasia after arterial injury.

### miR-22 Is Significantly Downregulated, Whereas Its Target Genes (MECP2 and EVI1) Are Dramatically Upregulated in the Diseased Human Arteries

**Figure 8.**
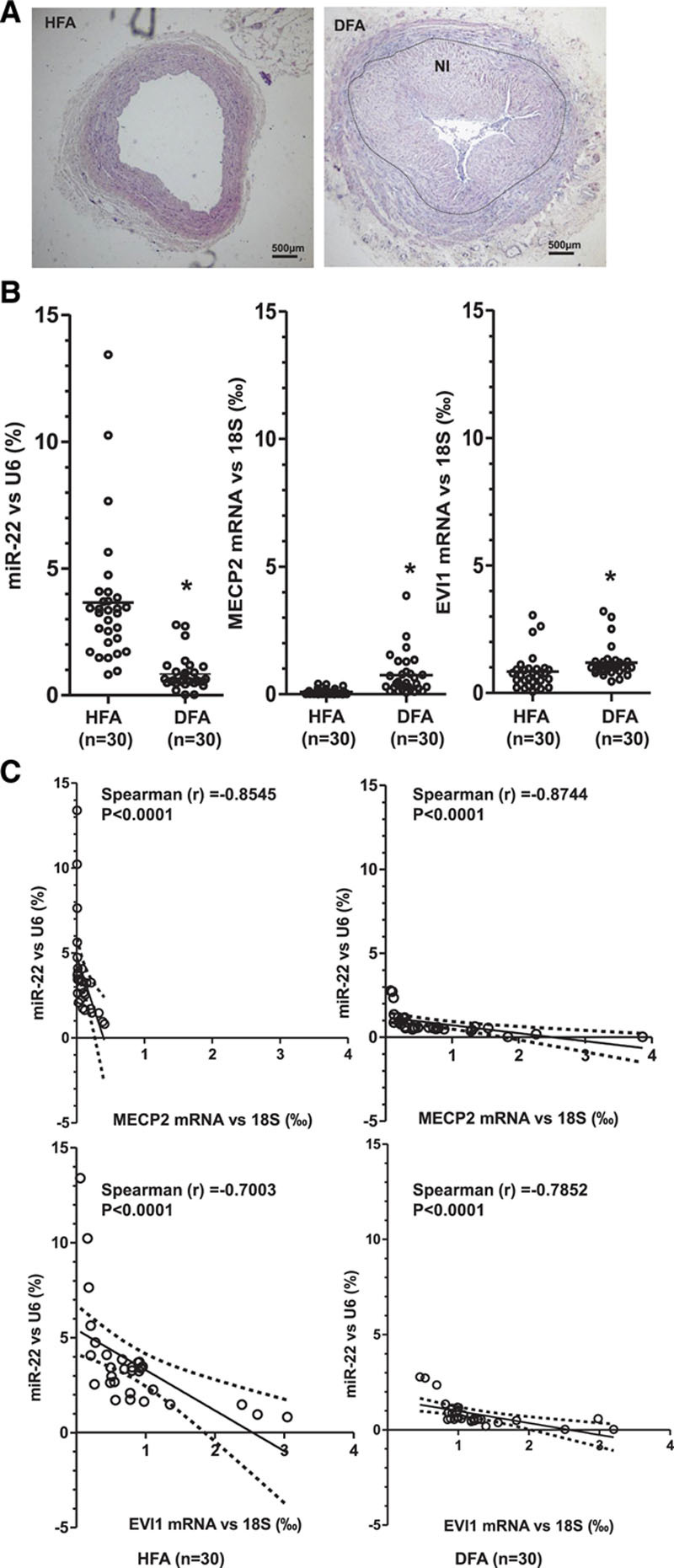
**Expression profiles of miR-22 and target genes in the healthy and diseased human arteries.** Healthy femoral artery specimens (HFA, n=30) from patients without peripheral arterial diseases and diseased femoral artery specimens (DFA, n=30) from patients with peripheral arterial diseases were collected and subjected to H&E staining (**A**) and RT-qPCR (**B**) analyses. **A**, Representative H&E images of HFA and DFA groups are shown. HFA is characterized by the absence of atherosclerotic lesions, whereas DFA is characterized by the presence of atherosclerotic lesions and severe neointima hyperplasia (NI). **B**, Expression levels of miR-22 (relative to U6, %) and its target genes, MECP2 (relative to 18S, ‰) and EVI1 (relative to 18S, ‰), are shown for each HFA and DFA specimen (dots). Lines represent the mean in each patient group. *P<0.05 (DFA versus HFA, Mann-Whitney *U* test). The expression level of miR-22 was significantly decreased, whereas the expression levels of MECP2 and EVI1 were dramatically increased in DFA. **C**, Spearman rank correlation analyses were performed to characterize the relationships between the gene expression levels of MECP2/EVI1 and miR-22 in HFA and DFA specimens. The *y* axis represents the expression level of miR-22 (relative to U6, %); the *x* axis represents the expression level of its target genes (MECP2 and EVI1) (relative to 18S, ‰). The solid line indicates the fitted linear regression line; the dotted line indicates 95% confidence interval level. *r* is the Spearman rank correlation coefficient between the expression levels of MECP2/EVI1 and miR-22. A value closer to –1 indicates a stronger negative correlation, and a value closer to 1 indicates a stronger positive correlation. *P* is the *P* value indicating the significant level of correlation. EVI1 indicates ecotropic virus integration site 1 protein homolog; H&E, hematoxylin and eosin; MECP2, methyl-CpG binding protein 2; miR-22, microRNA-22; and RT-qPCR, reverse transcription quantitative polymerase chain reaction.

To translate our findings from murine into human, we collected 30 diseased and 30 healthy femoral arterial specimens from patients who underwent leg amputation at the First Affiliated Hospital of Zhejiang University, China. Diseased femoral artery specimens featured SMC-rich atherosclerotic lesions with severe neointima hyperplasia, whereas healthy femoral arteries displayed no atherosclerotic lesions (Figure 8A). In comparison with healthy arteries, diseased femoral arteries showed a significantly decreased gene expression level of miR-22 and dramatically increased expression levels of its target genes (MECP2 and EVI1) (Figure 8B). It is more important that significant inverse relationships between miR-22 and its downstream targets, MECP2 and EVI1, were observed in both healthy and diseased femoral arteries (Figure 8C and Figure IX in the online-only Data Supplement). It is interesting to note that an abnormally high expression level of EVI1 was observed in 3 healthy and 3 diseased femoral arteries harvested from the patients with hypertension and hyperlipidemia, indicating that the coexistence of these 2 comorbidities could significantly affect EVI1 expression. Thus, these data provide critical information about the functional relevance of miR-22 and its target genes in human atherosclerotic lesions.

## Discussion

By using various in vivo, ex vivo, and in vitro models of VSMC phenotypic modulation, we identified a novel role of miR-22 as a mediator of VSMC phenotype switching and neointima formation. We specifically show that miR-22 is transcriptionally regulated by serum, PDGF-BB, and TGF-β1. Moreover, VSMC contractile gene expression, proliferation, and migration, but not apoptosis, were modulated by miR-22. Mechanistically, we have confirmed that MECP2, HDAC4, and EVI1 are the authentic downstream targets of miR-22 during VSMC phenotype switching. We have also identified EVI1 as a novel transcriptional repressor of VSMC contractile genes. It is important to note that we observed that miR-22 expression is suppressed in the human femoral arteries with atherosclerotic plaques and have uncovered an inverse relationship between miR-22 and its target genes, MECP2 and EVI1, in healthy and diseased arteries.

### Role of miR-22 in Heart Disease and Vascular Remodeling

miR-22 has been primarily identified as a tumor suppressor, but later studies have identified miR-22 as a prohypertrophic miR.^32,33,46,47^ A phenotypic screen with primary rat cardiomyocytes has suggested that miR-22 has prohypertrophic potential,^46^ which was further confirmed by using transgenic mice: specifically, global or cardiac-specific deletion of miR-22 blunted stress-induced cardiac hypertrophy and remodeling,^32,33^ whereas cardiac-specific overexpression of miR-22 induced a prohypertrophic gene expression profile and elicited cardiac dilation and heart failure.^47^ In a more clinically relevant study, pharmacological inhibition of miR-22 promoted cardiac functional recovery after myocardial infarction by eliciting cardiac autophagy.^30^ These important studies provide clear evidence to suggest that a fine balance of miR-22 expression and regulation is critical for maintaining adequate cardiac functions. Although the majority of miR-22 studies were conducted in cancer cells or cardiac cells, we recently reported an important role for miR-22 in VSMC differentiation from stem cells both in vitro and in vivo,^34^ inferring a regulatory role for miR-22 in VSMC biology. However, the role for miR-22 in VSMC phenotypic modulation had not been explored until our present study. By performing miR gain- and loss-of function studies, we demonstrated that miR-22 promotes VSMC contractile gene expression and inhibits VSMC proliferation and migration. We show that local transfer of miR-22 onto the injured arteries can restore synthetic VSMCs to a contractile phenotype, providing a basis for using site-specific delivery of miR-22 mimics via miR-22–coated stents to prevent or inhibit in-stent restenosis. Our study was also the first to identify an inverse relationship between the expression levels of miR-22 and its target genes, MECP2 and EVI1, in both healthy and diseased human femoral arteries, suggesting that miR-22 could be a potential therapeutic agent in coronary atherosclerosis. Therefore, extending our studies to other human arteries (eg, coronary/carotid arteries) would be a promising next step for exploring the therapeutic application of miR-22 in various cardiovascular diseases.

### Transcriptional Regulation of miR-22

The regulatory mechanisms of miR-22 expression in VSMCs are only partially known. Our previous publication suggested that miR-22 is transcriptionally regulated by PDGF-BB and TGF-β1 during VSMC differentiation from stem cells, but no direct evidence has been obtained.^34^ PDGF-BB is a member of the platelet-derived growth factor family primarily expressed by vascular endothelial cells and platelets at the vascular injury sites, and it is a key stimulant of VSMC proliferation and migration.^48,49^ In addition to upregulating miR-22 during VSMC differentiation from stem cells,^34^ PDGF-BB signaling has also been reported to transcriptionally induce miR-15b expression to mediate VSMC dedifferentiation and inhibit miR-221 to promote proliferation in pancreatic cancer cells.^50,51^ TGF-β1, initially identified as a tumor suppressor, is known to transcriptionally regulate genes involved in proliferation, growth, and differentiation by binding their response elements within target promoters.^52^ Similar to TGF-β1 signaling, the P53 signaling pathway can also modulate miR expression and maturation, and thereby cell proliferation and differentiation.^53^ A recent study showed that miR-22 expression is regulated by a P53-dependent mechanism during cardiac aging.^30^ In this study, we have provided definitive evidence that miR-22 gene expression in VSMCs is regulated by PDGF-BB and TGF-β1 through modulation of gene promoter activity (Figure 2C). Furthermore, our data also show that TGF-β1 transcriptionally regulates miR-22 in VSMCs via a P53-dependent mechanism. Our observation first establishes the TGF-β1/P53/miR-22 signaling axis and uncovers the regulatory role of PDGF-BB, TGF-β1, and P53 signaling pathways in VSMC phenotypic modulation. Because these major signaling pathways may represent novel therapeutic targets, the development of agents that target these signaling pathways is likely to have a significant therapeutic impact on vascular disease.

### EVI1 as Novel Gene Target of miR-22

Previous studies have shown that EVI1 functions as a transcriptional regulator that binds DNA sequences in the promoter region of target genes and regulates a number of biological processes, such as hematopoiesis, apoptosis, development, and cell differentiation and proliferation.^54–57^ However, little is known about its potential involvement in VSMC function and cardiovascular disease. In this study, we provide compelling evidence that EVI1 is the target gene of miR-22–mediated VSMC phenotype modulation and is a transcriptional repressor for multiple VSMC contractile genes. We specifically demonstrate that EVI1 transcriptionally inhibits VSMC-specific gene expression by providing the following evidence: (1) suppression of EVI1 expression and its reporter activity by miR-22 mimics; (2) increase in gene expression of all 5 VSMC-specific contractile markers and 2 transcription factors by EVI1 knockdown; (3) increase in gene promoter activity of SMαA, SM22α, SRF, and Myocd by EVI1 inhibition; (4) requirement of SRF binding sites(s) for EVI1-mediated SMαA and SM22α gene repression; and (5) direct binding and enrichment of EVI1 at the promoter regions of SMαA, SM22α, SRF, and Myocd confirmed by chromatin immunoprecipitation assay.

Emerging evidence has suggested that EVI1 regulates transcription, in part, through epigenetic modification.^56^ For example, the aberrant DNA hypermethylation signature in EVI1-directed acute myeloid leukemia is likely through interaction with DNA methyltransferases 3A and 3B.^58^ In this study, we observed a significant H3K9me3 enrichment within the promoter regions of VSMC-specific genes (SMαA, SM22α, SRF, and Myocd), and this enrichment was significantly inhibited by EVI1 knockdown (Figure 6F). Combined with the previous finding that another miR-22 target, MECP2, also modulates H3K9me3 enrichment within promoter regions of VSMC-specific genes,^34^ these results imply that epigenetic modification within VSMC gene promoters may be one of the underlying pathways through which miR-22 regulates VSMC phenotype switching. Whether other epigenetic mechanisms are involved in EVI1-mediated VSMC phenotype switching remains to be seen. Further investigations are warranted to identify genome-wide targets for EVI1 in VSMCs by conducting an unbiased EVI1 chromatin immunoprecipitation sequencing to better understand the global regulatory role of EVI1 in VSMC phenotype modulation and potentially in cardiovascular diseases.

It is noteworthy that our identification of the miR-22/EVI1 signaling axis in human atherosclerotic plaques presents potential clinical application toward treating cardiovascular disease. Because the expression of miR-22 was significantly decreased, whereas expression of its target genes, MECP2 and EVI, was dramatically increased in femoral atherosclerotic lesions in comparison with healthy femoral arteries, inhibiting EVI1 may offer a therapeutic opportunity to decrease neointima formation and restenosis. Furthermore, it has been reported that EVI1 protein can be specifically degraded by an anticancer drug, arsenic trioxide.^59^ Several preclinical^60,61^ and clinical^62,63^ studies suggest AES is a promising alternative to widely used sirolimus derivative–eluting stents. In a rabbit iliac artery injury model, AES significantly suppressed in-stent restenosis by reducing proliferation and inducing apoptosis of VSMCs.^61^ The beneficial effects of AES on in-stent restenosis in a porcine coronary model were also attributed to an early anti-inflammatory effect of arsenic trioxide in the stented vessels.^60^ A 2-year follow-up clinical study also demonstrated that AES has a comparable efficacy and safety to durable polymer sirolimus–eluting stent for the treatment of de novo coronary artery lesions.^62^ Our finding that the miR-22/EVI1 signaling axis plays an important role in VSMC phenotypic modulation and arterial remodeling may offer a possible mechanistic basis for the beneficial effect of AES on in-stent restenosis, and suggests that correcting the dysregulation of miR-22 and EVI1 in atherosclerotic arteries through a site-specific delivery of miR-22 mimics to the stented vessels by using a miR-22–coated balloon or stent, or ultrasound-triggered nanodelivery technology, could be a potential treatment to prevent or inhibit in-stent restenosis.

We recognize a few limitations of our study. First, we chose the mouse wire-injury model to study the therapeutic potential of miR-22 for treating postangioplasty restenosis, because it partially mimics balloon angioplasty and intraluminal stent placement, but further investigation using a stent model would increase the translational feasibility of our current findings. Another limitation is that this model fails to accurately emulate neointima formation attributable to native atherosclerosis, a major underlying cause of cardiovascular disease. Hence, extending our studies to a hyperlipidemia-induced atherogenic animal model is required to validate the therapeutic potential of miR-22 in other cardiovascular diseases.

Taken together, we present in this study compelling evidence that miR-22 is a novel regulator of VSMC phenotype switching and vascular neointima lesion formation, which acts via its target genes, MECP2, HDAC4, and EVI1. Our data have shown for the first time that ectopic expression of miR-22 in the injured arteries can reverse the process of VSMC phenotype switching and prevent postangioplasty restenosis, supporting a potential role for miR-22 and its target genes in a variety of proliferative vascular diseases. These findings may have extensive implications for the treatment of human atherosclerosis.

## Sources of Funding

This work was supported by the British Heart Foundation (FS/09/044/28007, PG/11/40/28891, PG/13/45/30326, PG/15/11/31279, PG/15/86/31723, and PG/16/1/31892 to Dr Xiao); the National Natural Science Foundation of China (91339102, 30900571, 81270001, 81570249, 91539103, and 81270180); and the Zhejiang Provincial Nature Science Foundation (LR14H020001 and LD18H020001). This work forms part of the research portfolio for the National Institute for Health Research Biomedical Research Center at Barts.

## Disclosures

None.

## Supplementary Material
