# Supplementary material for: miR-22 Is a Novel Mediator of Vascular Smooth Muscle Cell Phenotypic Modulation and Neointima Formation
Source: Circulation. 2018 Apr 23;137(17):1824–41. doi: 10.1161/CIRCULATIONAHA.117.027799 (PMC5916488; doi:10.1161/CIRCULATIONAHA.117.027799)
Supplement: Supplementary file 1 [file cir-137-1824-s001.pdf]

SUPPLEMENTAL MATERIAL

miRNA-22 is a novel mediator of vascular smooth muscle cell phenotypic modulation and neointima formation

Feng Yang[1,2#], Qishan Chen[1#], Shiping He[2], Mei Yang[1], Eithne Margaret Maguire[2], Weiwei An[2], Tayyab Adeel Afzal[2], Le Anh Luong[2], Li Zhang[1*] and Qingzhong Xiao[2,3,4*]

Supplemental Methods

Materials

Antibodies against MECP2 (rabbit IgG, ab2828) and EVI1 (rabbit, ab28457) were purchased from Abcam, UK. The antibody against α-tubulin (mouse) and all secondary antibodies were from Sigma. Other materials used in this study were purchased from Sigma unless specifically indicated otherwise.

Isolation and culture of thoracic aorta and smooth muscle cell (VSMC)

Thoracic aorta and primary murine VSMCs were isolated from mouse aorta and routinely maintained in DMEM supplemented with 10% serum (Fetal Bovine Serum, FBS) as described in our previous study[1]. Briefly, C57BL/6 mice (25-30 grams; Charles River Laboratories, UK) were euthanized by CO2, the thoracic aorta was dissected to remove adhering periadventitial tissue, and the endothelium was denuded with a catheter. The aortic tissues were either directly harvested for total RNA extraction (fresh aorta), cultured in DMEM containing 20% serum for 3 days (cultured aorta), or digested to obtain cultured VSMCs. Specifically, the aortic tissues were digested with Collagenase I solution (Sigma, 1 mg/ml) for 10 min at 37 °C followed by removing the adventitial layer, and the medial layer was minced into small pieces for second digestion with Collagenase I solution for 2 hours at 37 °C. After filtering with a Cell Strainer (70 µm), single cell digestion solution was centrifuged to remove the digestion solution. Cells were re-suspended in VSMC culture medium containing DMEM, 10% FBS, and 1% penicillin/streptomycin-glutamine and transferred into culture dishes pre-coated with 0.04% gelatin. Every batch of VSMCs was tested by immunofluorescence staining of smooth muscle cell (SMC) marker using a SMαA antibody (clone 1A4, 1:400; Sigma) to ensure that the purity of VSMCs was above 95%. Human aortic SMCs (hAoSMCs) were purchased from PromoCell GmbH (C-12533) and cultured in SMC growth medium 2 (PromoCell GmbH, C-22062) according to the manufacturer's instructions. Both murine and human VSMCs between passages 5 (P5) to P8 were treated with various stimuli as described in our previous studies[2, 3]. Briefly, for PDGF-BB and serum stimulation, VSMCs were grown to 80-90% confluence and serum-starved for 24~48 hours (0.5% FBS), followed by incubation with 20% FBS and 10ng/ml PDGF-BB for 3, 6, 12, 24, and 48 hours. For TGF-β1 treatments, VSMCs were serum-starved for 24~48 hours (0.5% FBS), followed by an incubation with 5ng/ml TGF-β1 for 24 or 48 hours.

Real time quantitative PCR (RT-qPCR) for mRNA and microRNAs

RT-qPCR was performed as previously described[2-6]. Briefly, total RNA containing small RNAs was extracted from murine aortas, or VSMCs using Trizol reagent (Sigma) according to the manufacturer's instructions. Total RNA containing small RNAs was extracted from human arterial specimens using miRCURY™ RNA Isolation Kits (FFPE) (EXIQON, 300115). RNAs were subjected to DNase I (Sigma) digestion to remove potential DNA contamination. Reverse transcription for long RNAs was performed using an Improm-II™ RT kit (Promega, Madison, WI, USA) with RNase inhibitor (Promega), and Random primers (Promega). The NCode™ VILO™ miRNA cDNA Synthesis Kit (Invitrogen, A11193-051) was used to synthesise poly (A) tails of all the miRNAs, followed by cDNA synthesis from the tailed population in a single reaction. The resultant cDNA was diluted to a working concentration of 5 ng/μl and stored at -20 ºC. NCode™ EXPRESS SYBR® GreenER™ qPCR SuperMix Universal was used in miRNA RT-qPCR. Relative mRNA or miRNA expression levels were normalized to 18S or U6 snRNA expression levels, respectively. Primers were designed using Primer Express software (Applied Biosystems), and the sequence for each primer was listed in **Supplemental Table 1**.

miR-22 mimics/inhibitor or siRNA transfection

Either miR-22 mimics, inhibitors, miRNA negative controls (Ctrl), control-scramble siRNA (si-NT), or MECP2-specific siRNA (si-MECP2) (25 nM, final concentration) were transfected into VSMCs using TransIT-X2 Transfection Reagent (Geneflow Limited, UK) according to the manufacturer's instructions. With Cy3™ Dye-Labeled Pre-miR Negative Control #1 (AM17120, Thermo Fisher Scientific Inc. UK), we detected over 70% of transfection efficiency for miRNA or siRNA transfection in VSMCs. All miRNA inhibitors or mimics and respective negative controls were purchased from Thermo Fisher Scientific Inc., and both control-scrambled siRNA and gene-specific siRNAs (MECP2 and HDAC4) were purchased from Sigma.

Functional assays for VSMCs

All functional studies to examine the effects of miR-22 and its target genes on VSMCs' behaviours were similarly conducted as described in our previous studies[1-3] and below.

VSMC proliferation assays

Cell counting

VSMCs were plated (3.5×10^4 per well) and cultured in 24-well plates pre-coated with 0.04% gelatin as described previously. Cells were allowed to grow to 80-90% confluence and transfected with miR-22 mimics, miR-22 inhibitor, MECP2 siRNA, or respective negative controls as indicated in the figures. Transfected cells were cultured overnight and subjected to serum starvation by culturing them in DMEM containing 0.5% serum for 24~48 hours. After starvation process, the cells were treated with 20% serum or PDGF-BB (10 ng/ml) for 48 hours before trypsinizing and manually counting the cells using a hematocytometer.

BrdU incorporation assay

VSMCs were transfected as described above, and were re-cultured (7.5×10^3 per well) in 96-well plates overnight, followed by serum starvation for 24 hours. Starved VSMCs were re-stimulated with 20% FBS or 10 ng/ml PDGF-BB, respectively, for

48 hours. Cell proliferation was evaluated using 5-Bromo-2'-deoxy-uridine (BrdU) Labeling and Detection Kit II (Roche) according to the manufacturer's instructions. Briefly, cells were incubated with BrdU at a final concentration of 10 μM for 12 hours before measurement. After fixation, cellular DNA was digested by nuclease and labelled with a peroxidase-conjugated BrdU antibody, followed by incubation with the peroxidase substrate. The absorbance of the samples was measured by a microplate reader at 405 nm (OD405) with reference measurement at 490 nm (OD490). Absorbance (A_{405nm}-A_{490nm}) values representing cell proliferation ability were compared between treatments.

VSMC migration assays

Wound healing (Scratch model)

Scratch wound healing assays were carried out using a previously described method[7]. In brief, VSMCs were cultured in 12-well plates and transfected with miR-22 mimics, miR-22 inhibitor, or respective miRNA negative controls (Ctrl) as described earlier. Transfected cells were allowed to grow to ~100% confluence and subjected to serum starvation for 24~48 hrs. After starvation process, the cells were treated with hydroxyurea (2 mM) to inhibit cell proliferation for 2 hours before subjecting them to 20% FBS or PDGF-BB (10 ng/ml) treatment. The cells were scratched in criss-cross manner and rinsed with PBS or DMEM three times to remove cell debris, before cultured in DMEM supplemented with 20% FBS or PDGF-BB (10 ng/ml) in the presence of 2 mM hydroxyurea. Denuded cell surface area of each wound at 0 hours (A0) and 24 hours (A24) was obtained by photomicrographic images and measured with ImageJ software by two experienced investigators who were blinded to the treatments. The percentages of cell closures (cell closure%) were calculated as (A0-A24)/A0*100.

Trans-well migration assay

VSMCs were cultured, transfected with miR-22 mimics, miR-22 inhibitor, MECP2 siRNA (si-MECP2), HDAC4 siRNA (si-HDAC4), or respective miRNA negative controls (Ctrl), and serum-starved as described earlier. Transfected cells were harvested by trypsinization. An aliquot (250,000 cells/200 µl) of the cells in serum-free DMEM was dispensed into the trans-well inserts (8 µm pore size, Greiner Bio-One Ltd, UK. Item number: 662638) pre-coated with 0.5% gelatin (Sigma, G1393), and DMEM with 20% serum or 30 ng/ml PDGF-BB was placed in the lower chamber. The trans-well plates were incubated at 37 °C in a 5% CO2 incubator for 12~18 hours. Non-migrated cells in the top insert were carefully removed by cotton swab, and the migrated cells in the bottom side were stained with Crystal Violet dye. Images were captured at five fixed locations (right, bottom, left, up, and centre), and migrated cells were counted by two experienced investigators blinded to the treatments.

Flow cytometry analysis for cell apoptosis

Similar to our previous study[2], VSMCs were transfected with miR-22 mimics, miR-22 inhibitor, or respective negative miRNA controls (Ctrl), and were subjected to various treatments as indicated. Cells were harvested and subjected to apoptosis

analyses using an Annexin V-FITC/PI kit (BMS306F1; Bender MedSystems) according to the manufacturer's instructions. After staining, cells were analysed using a FACSCalibur sorting system (Becton Dickinson). Cells with Annexin V^-/propidium iodide $(PI)^-$, Annexin V^+/PI^-, Annexin V^+/PI^+, or Annexin V^-/PI^+ were counted as live, early apoptotic, late apoptotic, or dead/necrotic cells, respectively.

Immunoblotting
Cells were harvested and sonicated in lysis buffer containing 50 mM Tris-Cl pH 7.5, 150 mM NaCl, 1 mM EDTA pH 8.0 and supplemented with protease inhibitors and 0.5% Triton. 40 μg of protein from cell lysate was separated by SDS-PAGE with 4%~20% Tris-Glycine gel (Invitrogen, Carlsbad, CA, USA) and subjected to standard Western blot analysis. Antibody to EVI1 (ab28457, 1:1000, Abcam); α-tubulin (T6074, 1:4000, Sigma), and MECP2 (ab2828, 1:1000, Abcam) were used to probe target proteins.

Transient transfection and luciferase assay
Luciferase assay for gene promoter or 3'UTR reporters were conducted as previous studies[2-6]. Briefly, for gene promoter activity assays, VSMCs were co-transfected with individual gene promoters (pGL3-miR-22, pGL3-SMαA, pGL3-SM22α, pGL3-SMαA-SRF^{mut}, pGL3-SM22α-SRF^{mut}, pGL3-SRF or pGL3-Myocd, the last six gene reporters were generated in our previous study[8]) (0.15 μg/$2.5x10^4$ cells) and p*Renilla* (15 ng/$2.5x10^4$ cells) using TransIT-X2 Transfection Reagent (Geneflow Limited, UK), according to the manufacturer's instructions. Transfected cells were subjected to various treatments as indicated in the respective figure legend. Dual-Luciferase Reporter Assay System was used for detecting luciferase and *Renilla* activities according to the protocol provided in the system. Relative luciferase unit (RLU) was defined as the ratio of Luciferase versus *Renilla* activity with that of the control (set as 1.0).
For MECP2 or EVI1 3'UTR reporter activity assays, VSMCs were co-transfected with individual reporter genes (pmiR-Luc-MECP2[9], pmiR-Luc-EVI1-WT, pmiR-Luc-EVI1-mutant, 0.15 μg/$2.5x10^4$ cells) and control or miR-22 mimics (25 nM) using TransIT-X2 Transfection Reagent (Geneflow Limited, UK), according to the manufacturer's instructions. pmiR-Luc-β-gal (0.20 μg/$2.5x10^4$ cells) was included in all transfection assays as internal control. Luciferase and β-galactosidase activities were detected 48 hours after transfection using a standard protocol. Relative luciferase unit (RLU) was defined as the ratio of Luciferase versus β-galactosidase activity with that of the control (set as 1.0).

Generation of EVI1 shRNA lentivirus and EVI1 stable knockdown VSMCs
EVI1 shRNA lentiviral particles were produced using MISSION shRNA EVI1 plasmids DNA (SHCLNG-NM_007963, MISSION® shRNA Bacterial Glycerol Stock, Sigma) according to protocol provided. The shRNA Non-Target control vector (SHC002) was used as a negative control (sh-NT). Briefly, 293T cells were transfected with the lentiviral vector and the packaging plasmids, pCMV-dR8.2 and pCMV-VSV-G (both obtained from Addgene), using TransIT-X2 Transfection Reagent (Geneflow Limited, UK) according to the manufacturer's instructions. The

supernatant containing the lentivirus was harvested 48 hours later, filtered, aliquoted, and stored at –80 °C. p24 antigen ELISA (Zeptometrix) was used to determine the viral titre. The Transducing Unit (TU) was calculated using the conversion factor recommended by the manufacturer (10^4 physical particles per pg of p24 and 1 transducing unit per 10^3 physical particles for a VSV-G pseudotyped lentiviral vector), with 1 pg of p24 antigen converted to 10 Transducing Units (TU). shRNA lentiviral infection and EVI1 stable knockdown VSMCs generation were performed as described in our previous studies with some modifications[8, 10, 11]. Briefly, VSMCs were plated 24 hours prior to infection in 6-well plates at 37 °C. One TU per cell (or 2-3x10^5/well) of shRNA or control virus was added with 10 μg/ml of hexadimethrine bromide (H9268; Sigma). Viral constructs were incubated for 24 hours with the cells before the media was replaced with complete media containing 4 μg/ml of puromycin (P9620, Sigma). For selection of transductants, fresh media containing puromycin was added at 2- to 3-day intervals for 7~10 days. Stably infected cells were split and frozen for future experiments.

Chromatin immunoprecipitation (ChIP) assays

The ChIP assays were performed as previously described[4, 8, 9, 12, 13]. Briefly, control (sh-NT) or EVI1 stable knockdown (sh-EVI1) VSMCs were treated with 1% (v/v) formaldehyde at room temperature for 10 minutes and then quenched with glycine. The medium was removed and the cells were harvested and sonicated. The sheared samples were diluted in 1 ml of immunoprecipitation buffer, and immunoprecipitation was conducted with antibodies raised against EVI1 (rabbit, ab28457), H3K9me3 (mouse, 05-1250), or respective IgG controls (5μg/immunoprecipitation). Immunoprecipitation complexes were pulled down using protein-G Dynabeads. The immunoprecipitates were eluted from the beads using 50 μl of elution buffer, and immunoprecipitaed DNA was extracted, purified, and then used to amplify target DNA sequences by RT-qPCR using specific primers (Online **Supplemental Table 1**). Promoter DNA enrichment with specific antibodies was calculated using percent input method with that of the IgG control set as 1.0. PCR amplification of the adjacent promoter regions (for SRF and Myocd) or regions lacking SRF binding sites (without CArG region) (for SMαA and SM22α) were included as an additional control for specific promoter DNA enrichment. The data was obtained from three to four independent experiments.

Mouse femoral artery denudation injury and perivascular delivery of miR-22 agomiRs or LNA-miR-22

Anesthetized C57BL/6 mice underwent surgical procedure as described previously[1, 2, 14, 15]. Wire-induced denudation injury was achieved by removing the endothelium of the femoral arteries with 3~5 passages of a 0.25 mm angioplasty spring-wire (Tips of cross-IT 200x guide wire, Abbott Laboratories. Illinois, USA). For perivascular delivery of miR-22, wire-injured femoral arteries were randomly assigned miR-22 or Cel-miR-67 agomiRs group and applied with pluronic gel as described in our previous studies[2, 3]. Briefly, after wire injury, 100 μl of 30% pluronic gel containing chemically modified and cholesterol-conjugated 2.5 nmol miR-22 or scrambled (Cel-miR-67) agomiRs was applied perivascularly to injured femoral arteries. The micrON™

miRNA agomiRs were purchased from RiboBio (Guangzhou RiboBio Co., Ltd., China); the *in vivo* expression efficiency and stability of such agomiRs has been extensively demonstrated by many research groups worldwide[16-19].
For miR-22 knockdown experiment, 100 µl of 30% pluronic gel containing vehicle (mock transfection), control LNA (Scrambled LNA), or LNA-miR-22 per vessel per mouse was applied perivascularly to injured femoral arteries. Locked nucleic acid (LNA) modified antisense (LNA-miR-22) and scrambled control oligonucleotide (Scrambled LNA) were purchased from EXIQON with their corresponding sequences -5'- CAGTTCTTCAACTGGCAGCT-3' and -5'-ACGTCTATACGCCCA-3'.

Additional femoral arteries were harvested and dissociated at three (agomiRs) or seven (LNA-miRNAs) days after injury. Five femoral arteries from each group were pooled for each independent experiment and triplicate experiments were conducted. Total RNAs (including small RNAs) were extracted for RT-qPCR of miR-22 and mRNAs of genes of interest. All animal experiments were performed according to protocols approved by the Institutional Committee for Use and Care of Laboratory Animals at Queen Mary University of London, UK.

Morphometric analysis and quantification of neointima formation
The mouse femoral arteries were harvested four weeks after the operation. The specimens were fixed in 4% formaldehyde and subjected to paraffin embedding and sectioning processes. Sections (8µm) were collected at 100µm intervals (10 sections per segment/interval), mounted on slides, and numbered. Six digitised sections with the same identification number from three segments/intervals (~0.4mm, 0.5mm, and 0.6mm from injury site) of each animal (e.g. IV-1/2, V-1/2, and VI-1/2 represent the 1st and 2nd section of the 4th, 5th and 6th segment/interval, respectively) were stained with H&E for morphometric analysis. The procedure for lesion quantification was similar to previous descriptions[1-3, 14, 15]. Briefly, the area of media and intima on cross-section of H&E-stained artery segments were automatically measured in pixel-squared ($pixel^2$) with a computerized image analysis system (Axiovision software) by two experienced investigators blinded to the treatments.

Supplemental References

1. Xiao Q, Zhang F, Grassia G, Hu Y, Zhang Z, Xing Q, Yin X, Maddaluno M, Drung B, Schmidt B, Maffia P, Ialenti A, Mayr M, Xu Q, Ye S. Matrix metalloproteinase-8 promotes vascular smooth muscle cell proliferation and neointima formation. *Arteriosclerosis, thrombosis, and vascular biology*. 2014;34:90-98
2. Chen Q, Yang F, Guo M, Wen G, Zhang C, Luong le A, Zhu J, Xiao Q, Zhang L. Mirna-34a reduces neointima formation through inhibiting smooth muscle cell proliferation and migration. *Journal of molecular and cellular cardiology*. 2015;89:75-86
3. Afzal TA, Luong LA, Chen D, Zhang C, Yang F, Chen Q, An W, Wilkes E, Yashiro K, Cutillas PR, Zhang L, Xiao Q. Nck associated protein 1 modulated by mirna-214 determines vascular smooth muscle cell migration, proliferation, and neointima hyperplasia. *Journal of the American Heart Association*. 2016 Dec 7;5(12). pii: e004629.
4. Luo Z, Wen G, Wang G, Pu X, Ye S, Xu Q, Wang W, Xiao Q. Microrna-200c and -150 play an important role in endothelial cell differentiation and vasculogenesis by targeting transcription repressor zeb1. *Stem Cells*. 2013;31:1749-1762
5. Yu X, Zhang L, Wen G, Zhao H, Luong LA, Chen Q, Huang Y, Zhu J, Ye S, Xu Q, Wang W, Xiao Q. Upregulated sirtuin 1 by mirna-34a is required for smooth muscle cell differentiation from pluripotent stem cells. *Cell Death Differ*. 2015 Jul;22(7):1170-1180.
6. Zhao H, Wen G, Huang Y, Yu X, Chen Q, Afzal TA, Luong LA, Zhu J, Shu Y, Zhang L, Xiao Q. Microrna-22 regulates smooth muscle cell differentiation from stem cells by targeting methyl cpg-binding protein 2. *Arteriosclerosis, thrombosis, and vascular biology*. 2015 Apr;35(4):918-929.
7. Liang CC, Park AY, Guan JL. In vitro scratch assay: A convenient and inexpensive method for analysis of cell migration in vitro. *Nat Protoc*. 2007;2:329-333
8. Huang Y, Lin L, Yu X, Wen G, Pu X, Zhao H, Fang C, Zhu J, Ye S, Zhang L, Xiao Q. Functional involvements of heterogeneous nuclear ribonucleoprotein a1 in smooth muscle differentiation from stem cells in vitro and in vivo. *Stem cells*. 2013;31:906-917
9. Zhao H, Wen G, Huang Y, Yu X, Chen Q, Afzal TA, Luong le A, Zhu J, Ye S, Zhang L, Xiao Q. Microrna-22 regulates smooth muscle cell differentiation from stem cells by targeting methyl cpg-binding protein 2. *Arteriosclerosis, thrombosis, and vascular biology*. 2015;35:918-929
10. Xiao Q, Zhang F, Lin L, Fang C, Wen G, Tsai TN, Pu X, Sims D, Zhang Z, Yin X, Thomaszewski B, Schmidt B, Mayr M, Suzuki K, Xu Q, Ye S. Functional role of matrix metalloproteinase-8 in stem/progenitor cell migration and their recruitment into atherosclerotic lesions. *Circulation research*. 2013;112:35-47

11. Fang C, Wen G, Zhang L, Lin L, Moore A, Wu S, Ye S, Xiao Q. An important role of matrix metalloproteinase-8 in angiogenesis in vitro and in vivo. *Cardiovascular research*. 2013;99:146-155
12. Xiao Q, Pepe AE, Wang G, Luo Z, Zhang L, Zeng L, Zhang Z, Hu Y, Ye S, Xu Q. Nrf3-pla2g7 interaction plays an essential role in smooth muscle differentiation from stem cells. *Arterioscler Thromb Vasc Biol*. 2012;32:730-744
13. Wang G, Xiao Q, Luo Z, Ye S, Xu Q. Functional impact of heterogeneous nuclear ribonucleoprotein a2/b1 in smooth muscle differentiation from stem cells and embryonic arteriogenesis. *J Biol Chem*. 2012;287:2896-2906
14. Xiao Q, Zeng L, Zhang Z, Margariti A, Ali ZA, Channon KM, Xu Q, Hu Y. Sca-1+ progenitors derived from embryonic stem cells differentiate into endothelial cells capable of vascular repair after arterial injury. *Arteriosclerosis, thrombosis, and vascular biology*. 2006;26:2244-2251
15. Zeng L, Xiao Q, Margariti A, Zhang Z, Zampetaki A, Patel S, Capogrossi MC, Hu Y, Xu Q. Hdac3 is crucial in shear- and vegf-induced stem cell differentiation toward endothelial cells. *The Journal of cell biology*. 2006;174:1059-1069
16. Cai J, Yang C, Yang Q, Ding H, Jia J, Guo J, Wang J, Wang Z. Deregulation of let-7e in epithelial ovarian cancer promotes the development of resistance to cisplatin. *Oncogenesis*. 2013;2:e75
17. Wang X, Guo B, Li Q, Peng J, Yang Z, Wang A, Li D, Hou Z, Lv K, Kan G, Cao H, Wu H, Song J, Pan X, Sun Q, Ling S, Li Y, Zhu M, Zhang P, Peng S, Xie X, Tang T, Hong A, Bian Z, Bai Y, Lu A, He F, Zhang G. Mir-214 targets atf4 to inhibit bone formation. *Nat Med*. 2013;19:93-100
18. Krutzfeldt J, Rajewsky N, Braich R, Rajeev KG, Tuschl T, Manoharan M, Stoffel M. Silencing of micrornas in vivo with 'antagomirs'. *Nature*. 2005;438:685-689
19. Trajkovski M, Hausser J, Soutschek J, Bhat B, Akin A, Zavolan M, Heim MH, Stoffel M. Micrornas 103 and 107 regulate insulin sensitivity. *Nature*. 2011;474:649-653

Supplemental Tables

Supplemental Table 1: Primer sets used in the present study

Gene names	Forward (5'-3')	Reverse (5'-3')	Application
U6 snoRNA (mus/hu)	gatgacacgcaaattcgtg	miRNA universal reverse primer (Invitrogen, A11193-051)	Real-time RT-PCR
miR-22 (mus/hu)	AAGCTGCCAGTTGAAGAACTGT	miRNA universal reverse primer (Invitrogen, A11193-051)	Real-time RT-PCR
18s (mus/hu)	aaacggctaccacatccaag	cctccaatggatcctcgtta	Real-time RT-PCR
Pri-miR-22 (mus)	AAAGGGGCACAAAGCAAGTG	CAGGAAAGCTGGGTGACAGG	Real-time RT-PCR
Pre-miR-22 (mus)	ACCTGGCTGAGCCGCAGTAG	AGGGGCAGAGGGCAACAGTTC	Real-time RT-PCR
Mus SMαA	TCCTGACGCTGAAGTATCCGAT	GGCCACACGAAGCTCGTTATAG	Real-time RT-PCR
Mus SM22α	GAT ATG GCA GCA GTG CAG AG	AGT TGG CTG TCT GTG AAG TC	Real-time RT-PCR
Mus CNN1	GCGTCACCTCTATGATCCCAA	CCCAGACCTGGCTCAAAGAT	Real-time RT-PCR
Mus SM-myh11	AAG CAG CCA GCA TCA AGG AG	AGC TCT GCC ATG TCC TCC AC	Real-time RT-PCR
Mus SMTN-B	GGGCAGTATCTTCGACCGAG	GGCAGACTCTGTGCCTTCAT	Real-time RT-PCR
Mus MECP2	GGCTGTGGTAAAACCCGTCCG	GGCTTGTCTCTGAGGCCCTGGA	Real-time RT-PCR
Mus EVI1	GCAGACATTGCGCCTGGGGAA	CTCACAGCGGTGCTGCCGTT	Real-time RT-PCR
Mus SRF	CCTACCAGGTGTCGGAATCTGA	TCTGGATTGTGGAGGTGGTACC	Real-time RT-PCR
Mus Myocd	TCAATGAGAAGATCGCTCTCCG	GTCATCCTCAAAGGCGAATGC	Real-time RT-PCR
Mus MEF2C	AAGCCAAATCTCCTCCCCCTAT	TGATTCACTGATGGCATCGTGT	Real-time RT-PCR
Mus PCNA	TTGCACGTATATGCCGAGACCT	ATTGCCAAGCTCTCCACTTGC	Real-time RT-PCR
Mus TET2	CCATCATGTTGTGGGACGGA	TCTGAGAACAGCGACGGTTG	Real-time RT-PCR
Mus RAB5B	GGAGCCCCAGGACTGTTGA	CCTGGCTGTACTTCTGCTAGT	Real-time RT-PCR
Mus Trp53inp1	ATGCTGTGCACAACTCCTGT	TCTGGGCTTCCATTCTGGGA	Real-time RT-PCR
Mus AKT3	GGTGCAGAGTCCCCTAGAGA	TTGGCGACAGCAGGATCATT	Real-time RT-PCR
Mus SirT1	GCTCGCCTTGCGGTGGACTT	GACGGCTGGAACTGTCCGGG	Real-time RT-PCR
Mus ARPC5	GTGGGGTCCATTGTTCGAGT	TGAACGGTGTCCAGTTCCAG	Real-time RT-PCR
Mus PTEN	AGCCTCTTGATGTGTGCATT	CCATTGGTAGCCAAACGGAAC	Real-time RT-PCR
Mus ADAM11	CAGCAACGAGGGGAAGTGTA	ATGATGTTGGTACCGCTGGG	Real-time RT-PCR
Mus PPARα	GAGGGCACACGCTAGGAAG	CGAACACCAATGTTCGGAGC	Real-time RT-PCR
Mus Arhgef12	CCCAGATAGAGCGGGGACC	ACTTCGTCACAGAGGGAAGTC	Real-time RT-PCR
Mus MAP3K3	ATCGCCACCATGGATGAACA	ACTCTGCCTGTTTGGGTGAC	Real-time RT-PCR
Mus TGFβR1	AAGCATTGGCAAAGGTCGGT	CGGAACCATGAACGCTCTTCT	Real-time RT-PCR
Mus P21	GGAGGCCCGAGAACGGTGGA	CAGCCCTAGGCTCCGAACGC	Real-time RT-PCR
Mus KAT5	AGTGGAGGGAGGGAAGATGG	CTTTCGGCCACTGATGTCCT	Real-time RT-PCR
Mus MAP2K4	TTCTGTGAAAAGGCACAAAGTAAG	TCTCAGTCTCTCTATGTGTGGGT	Real-time RT-PCR
Mus CDC25a	AGAAGCTCCTGGGATGTAGC	TGAGGTGAAAGGTGTCGTGG	Real-time RT-PCR
Mus Myst4	CGGGCTGGAGGCGAGGTCTT	TGACTGCCAGGCAACGTGGA	Real-time RT-PCR
Mus PDAP1	CAAAAGCGAAAAGGCGTGGA	CAGATCCAGTTGCGTGACCT	Real-time RT-PCR
Mus Sp1	ACAGGGTGCCAATGGCTGGC	GCCCACCAGAGACTGTGCGG	Real-time RT-PCR
Mus HDAC4	TGCGTCCTGCCTTGGTGCTC	GCGCGCTGTCCTGTGTTCCA	Real-time RT-PCR
Mus	TGGATATTTGGTCCGTGGGC	TTCTTCAGAAGCTCAGCCCC	Real-time RT-PCR

MAPK14			
hu SMαA	TGAGCGTGGCTATTCCTTCGT	GCAGTGGCCATCTCATTTTCAA	Real-time RT-PCR
hu SM22α	GGCTGAAGAATGGCGTGATT	TCTGCTTGAAGACCATGGAGG	Real-time RT-PCR
hu SM-myh11	GCGTCCATGCCAGATAACACA	TACCACATCTCGCCCAACCTT	Real-time RT-PCR
hu SMTN-B	TGGAGTCCATGAACGATGTGG	TCAATCTCCTGAGCCCGTACAC	Real-time RT-PCR
Hu MECP2	ATGTGACATGTGACTCCCCA	TTTCTTCCCTGAGCCCTAACA	Real-time RT-PCR
Hu EVI1	GGCTAGATTGCTTATTCATAGGGC	AATGTACTTGAGCCAGCTTCC	Real-time RT-PCR
SMαA promoter	CATAACGAGCTGAGCTGCCTC	CCAAACAAGGAGCAAAGACG	CHIP assay
SMαA adjacent	GATCAGAGCAAGGGGCTATA	CTACTTACCCTGACAGCGAC	CHIP assay
SM22α promoter	GCAGGTTCCTTTGTCGGGCCA	CTGCTTGGCTCACCACCCCG	CHIP assay
SM22α adjacent	CTTTAAACCCCTCACCCAGC	ATGACTTGCACTTACAAGG	CHIP assay
SRF promoter	GGCTGGGCCCTCCCCCATTT	TGGCTGGTTTGCTGGTTTGGCA	CHIP assay
SRF adjacent	CCCTCTTCTGCCCTGCAGTCCT	CCGCGATTCCGTGGGAGGGA	CHIP assay
Myocd promoter	CCCGCTCACCGCTCCTGATT	AGCCGCATCCTAGGCGTTCCT	CHIP assay
Myocd adjacent	CGGGAGTTGCAAGCCAACCCA	TCCCCAGCTTACTGCAGGGCT	CHIP assay
pmiR-Luc-EVI1-WT	GACCTG GAGCTC GTTGACCAGAGTGGGACCAAGTCCA	CAGCAG ACGCGT AACATCATCGCACAAGCTTGCAGA C	EVI1 3'UTR reporter clone
pmiR-Luc-EVI1-BS^{mu}	ttgcacagccaa TTTGAAG gctgacttctgg	ccagaagtcagc CTTCAAA ttggctgtgcaa	miR-22 binding site mutation
pGL3-miR-22-Luc	GTGGTG GGTACC TGCCCGAGCTGACCATTTAG	GTGGTG ACGCGT GGGTTAGAAATGGGAGGGGC	miR-22 gene promoter clone

Supplemental Table 2: Baseline Patient Characteristics

Patient Characteristic	Healthy femoral artery group (HFA) (n=30)	Diseased femoral group (DFA) (n=30)	P value
**Demographics**			
Female sex, no. (%)	7 (23.3)	8 (26.7)	0.776‡
Age (years)	55.83 ± 14.05	61.97 ± 14.65	0.103*
**Clinical Parameters**			
Current smoker, no. (%)	5 (16.67)	9 (30)	0.361‡
Former smoker, no. (%)	1 (3.33)	8 (26.67)	0.026‡
BMI, kg/m^2	23.04 ± 3.51	21.76 ± 3.62	0.201*
SBP, mmHg	137.9 ± 25.53	122.23 ± 19.11	0.009*
DBP, mmHg	75.87 ± 12.85	74.77 ± 13.05	0.743*
Heart rate, beats per minute	88.67 ± 15.66	84.6 ± 15.05	0.309*
**Comorbidities**			
Prior stroke, no. (%)	1 (3.33)	4 (13.33)	0.353‡
Carotid artery disease, no. (%)	2 (6.67)	2 (6.67)	1‡
Coronary artery disease, no. (%)	1 (3.33)	2 (6.67)	1‡
Hypertension, no. (%)	6 (20)	21 (70)	0.0001‡
Type II diabetes mellitus, no. (%)	0 (0)	12 (40)	NA
Hyperlipidaemia, no. (%)	4 (13.33)	22 (73.33)	<0.0001‡
Chronic kidney disease, no. (%)	0 (0)	8 (26.67)	NA
**Medications**			
Aspirin, no. (%)	1 (3.33)	25 (83.33)	<0.0001‡
Statin, no. (%)	0 (0)	21 (70)	NA
ACEI/ARB, no. (%)	3 (10)	9 (30)	0.104‡
Beta blockers, no. (%)	0 (0)	6 (20)	NA
Clopidogrel, no. (%)	0 (0)	6 (20)	NA
Cilostazol, no. (%)	0 (0)	9 (30)	NA
Other antihypertensive medications, no. (%)	3 (10)	17 (56.67)	0.0003‡
Other antithrombotic medications, no. (%)	2 (6.67)	28 (93.33)	<0.0001‡
**Laboratory Findings**			
eGFR, ml/minute	106.01 ± 41.41	84.72 ± 46.88	0.072†
hsCRP, mg/L	58.19 ± 46.55	103.05 ± 55.03	0.0182†
Total cholesterol, mM/L	3.10 ± 1.03	3.715 ± 1.127	0.034*
Triglycerides, mM/L	1.56 ± 1.06	1.253 ± 0.53	0.167*
HDL cholesterol, mM/L	0.79 ± 0.38	0.88 ± 0.36	0.366*
LDL cholesterol, mM/L	1.55 ± 0.88	2.03 ± 0.92	0.0486*
Fasting plasma glucose, mM/L	5.06 ± 0.67	7.62 ± 4.69	0.009*

Leukocytes, $\times 10^9$/L	8.62 ± 3.03	11.91 ± 5.43	0.005†
Hemoglobin, g/L	95.57 ± 27.18	112.33 ± 33.52	0.05*

P value is calculated with t-test (*), Mann-Whitney U Test (†) or Fisher's exact test (‡) to compare continuous variables (presented as mean ± SD) or categorical variables (presented as no. (%)), respectively.

SMC, smooth muscle cell; SD, standard deviation; BMI, body mass index; SBP, systolic blood pressure; DBP, diastolic blood pressure; ACEI/ARB, angiotensin-converting enzyme inhibitor/angiotensin receptor blocker; eGFR, estimated glomerular filtration rate; hsCRP, high-sensitivity C-reactive protein; HDL, high-density lipoprotein cholesterol; LDL, low-density lipoprotein cholesterol; NA, not applicable.

Body mass index was computed as the weight in kilograms divided by the square of the height in meters.

For the measurement of blood pressure, the patient first rested 5 minutes in a seated position. Blood pressure was then measured 3 separate times and the mean of the last 2 values was used. A systolic blood pressure ≥140 mmHg, and/or a diastolic blood pressure ≥90 mmHg was defined as hypertension; moreover, a patient had specific prescription records of anti-hypertensive drugs or currently taking anti-hypertensive medications was also defined as hypertension.

Diabetes was defined as a fasting glucose value ≥7.0mM/L or a history of diabetes medication use.

Prior stroke was defined as an episode of neurological dysfunction caused by focal cerebral, spinal, or retinal infarction.

Carotid/coronary artery disease was defined as a maximal arterial luminal stenosis ≥70% in the indexed arteries measured by digital subtraction angiography or other non-invasive methods, such as duplex ultrasound, computed tomography angiography, or magnetic resonance angiography.

Chronic kidney disease was defined as either kidney damage or a decreased eGFR of less than 60 mL/min/1.73 m^2 for at least 3 months.

Hyperlipidaemia was defined as an abnormally elevated level of at least one lipid or lipoprotein in the blood (total cholesterol ≥6.2mM/L, triglycerides ≥2.3mM/L, and/or LDL cholesterol ≥4.1mM/L); An individual with specific prescription records of lipid-lowering agents or currently taking lipid-lowering medications is also considered to have hyperlipidaemia.

Medications was self-reported by patients or prescribed by the surgeons.

Supplemental Figures and Figure Legends

Supplemental Figure 1. miR-22 controls VSMC marker gene expression.

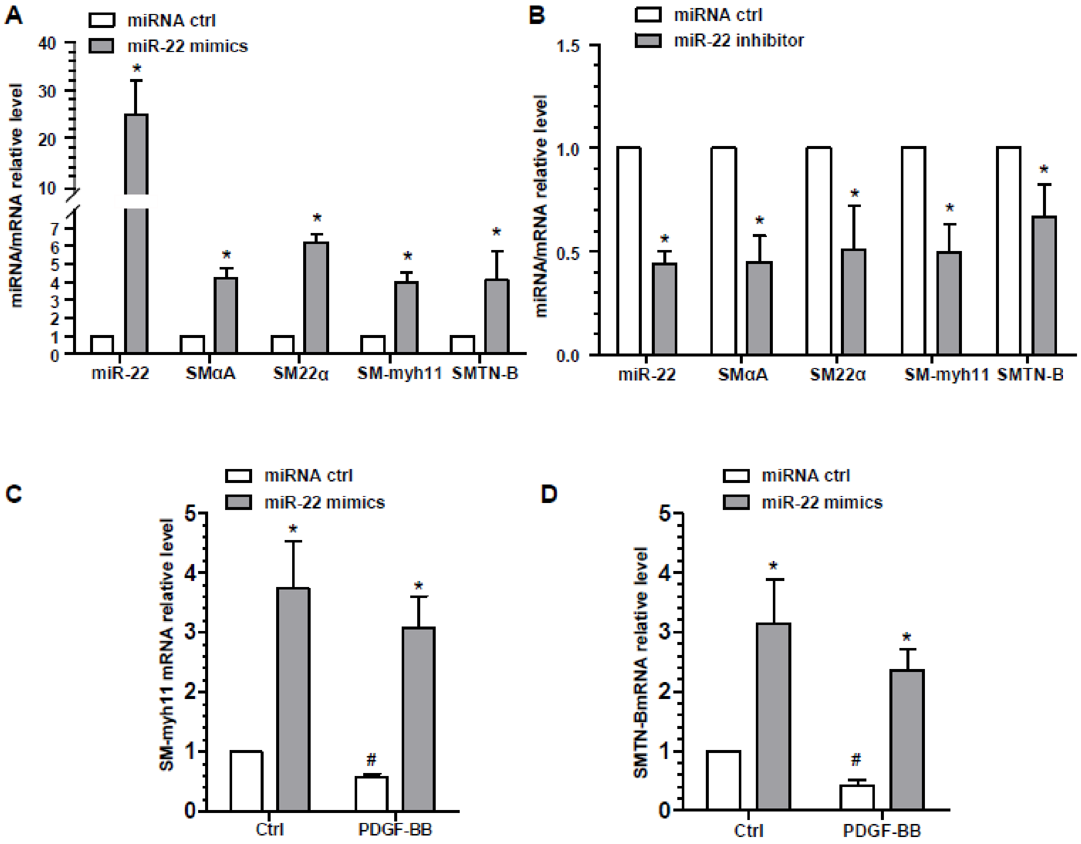

(A-B) miR-22 over-expression increases while miR-22 inhibition decreases VSMC marker gene expression. VSMCs were transfected with miR-22 mimics (A), a miR-22 inhibitor (B), or respective control miRNAs (miRNA ctrl), followed by 48 hours of serum starvation. (C-D) miR-22 over-expression prevents PDGF-BB-induced VSMC gene repression. VSMCs transfected with miR-22 mimics or control miRNAs (miRNA ctrl) were subjected to serum starvation for 48 hours, followed by PDGF-BB (10 ng/ml) stimulation for 6 hours. Total RNAs were harvested and subjected to RT-qPCR analyses with indicated primers. Data presented here are averaged from three to four independent experiments (n=3 in A, C, and D; n=4 in B). *P<0.05 (versus miRNA ctrl), #P<0.05 (versus control treatment).

Supplemental Figure 2. miR-22 regulates VSMC growth and migration.

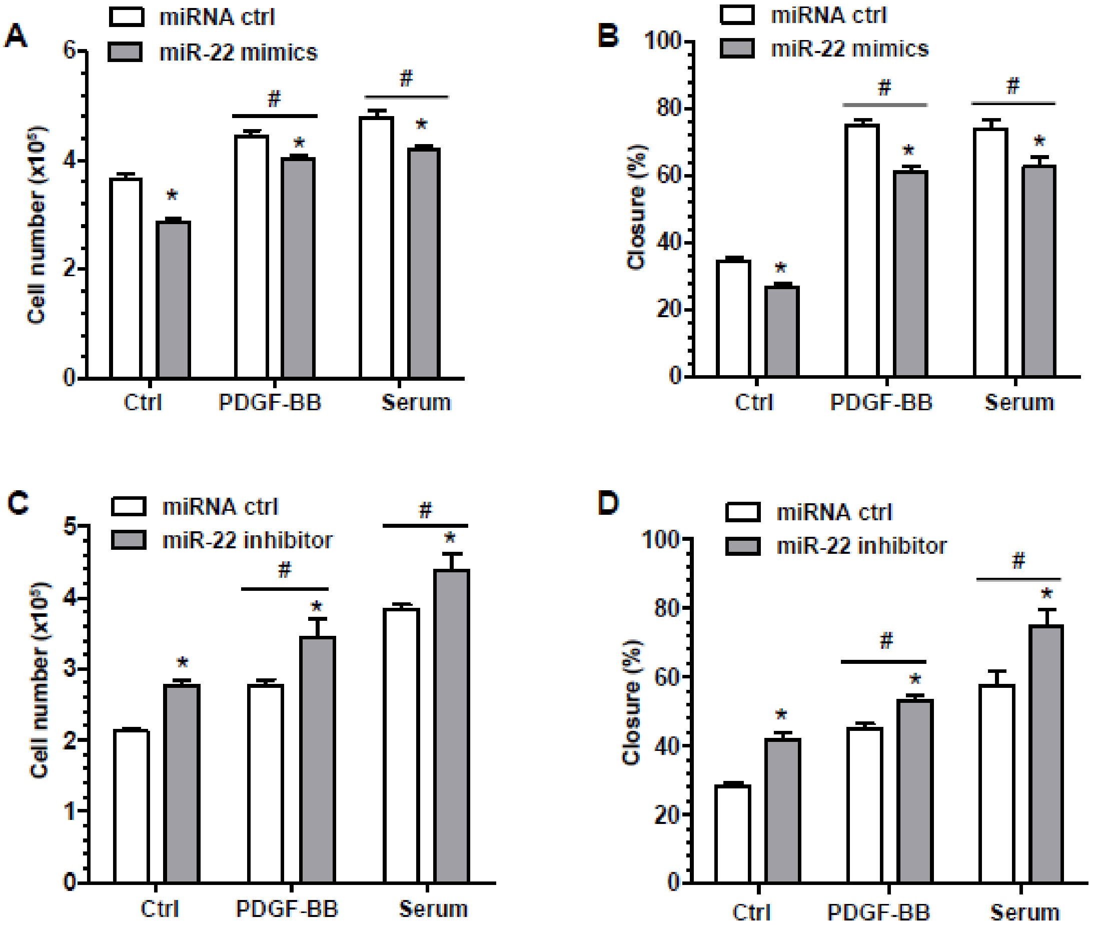

VSMCs were transfected with miR-22 mimics (A-B), a miR-22 inhibitor (C-D), or respective control miRNAs (miRNA ctrl), followed by 24 hours of serum starvation. Subsequently, cells were treated with PDGF-BB (10 ng/ml) or 20% serum for a further 48 hours, followed by cell counting (A and C) and wound-healing assays (B and D). In wound-healing assays, the percentage of cell closure (%) was calculated as described in the method section. Data presented here are averaged from three to four independent experiments (n=3 in A, C, and D; n=4 in B). *P<0.05 (versus miRNA ctrl), #P<0.05 (versus Ctrl).

Supplemental Figure 3. miR-22 modulates VSMC marker gene expression, proliferation, and migration in human aortic SMCs.

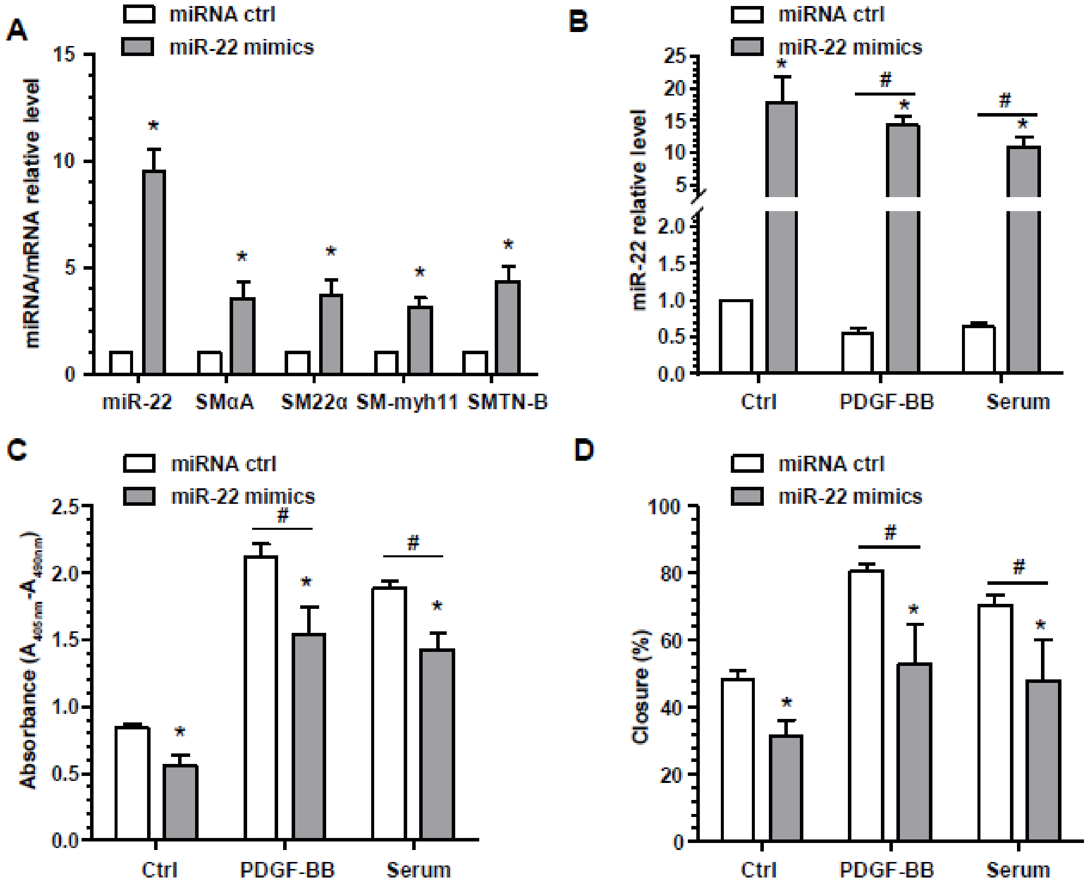

Human aortic SMCs (hAoSMCs) transfected with miR-22 mimics or control miRNAs (miRNA ctrl) were subjected to serum starvation for 48 hours. Subsequently, cells were harvested for RT-qPCR to examine gene expression levels (A). RT-qPCR (B), BrdU incorporation (C) and wound-healing (D) assays were performed to measure the miR-22 expression level, proliferation and migration of serum-starved hAoSMCs in response to PDGF-BB (10 ng/mL) or 20% serum stimulation. In wound-healing assays, the percentage of cell closure (%) was calculated as described in the method section. Data presented here are averaged from three to five independent experiments (n=3 in A; n=4 in B, C, and E; n=5 in D and F). *P<0.05 (versus miRNA ctrl), #P<0.05 (versus Ctrl).

Supplemental Figure 4. miR-22 plays no role in VSMC apoptosis.

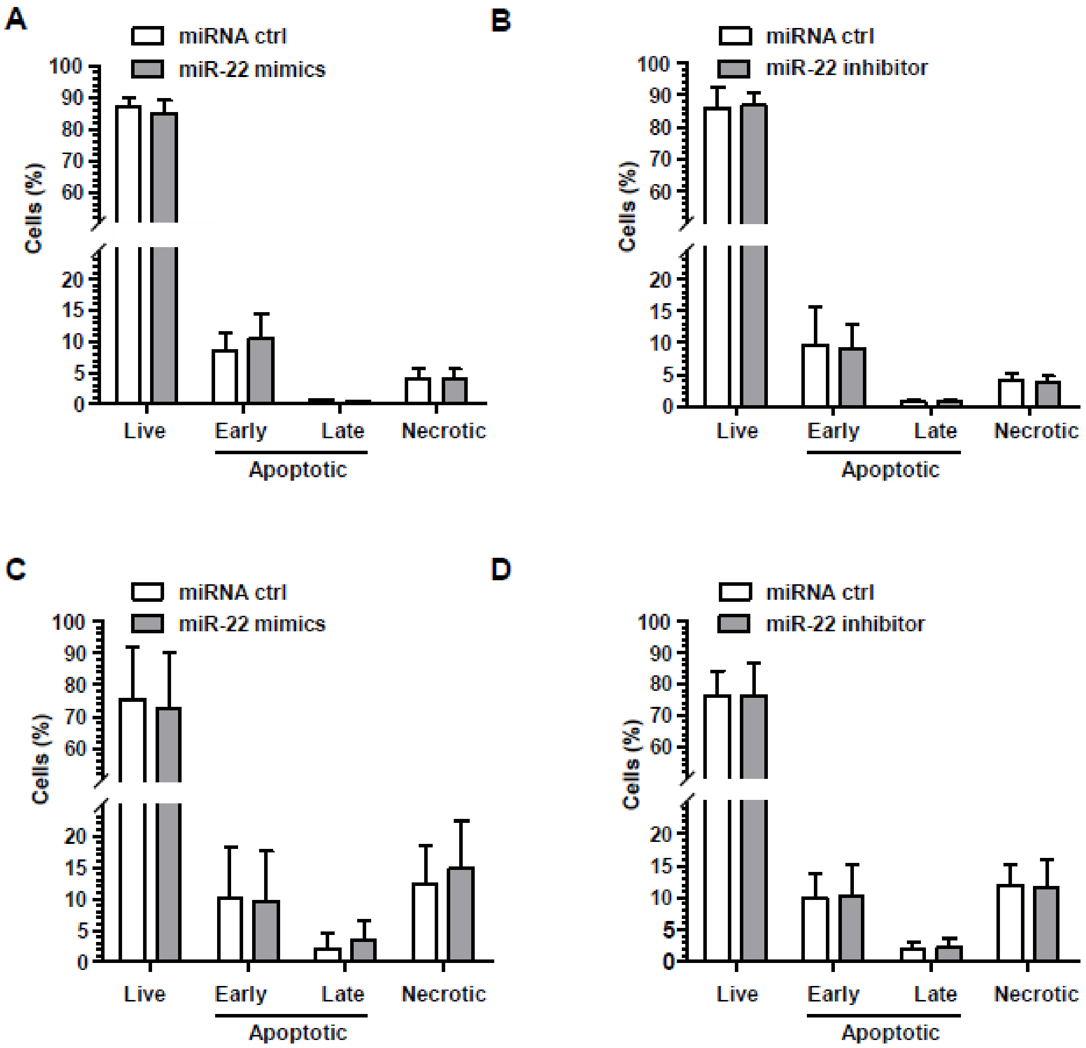

VSMCs were transfected with miR-22 mimics (A and C), a miR-22 inhibitor (B and D), or respective control miRNA (miRNA ctrl) as indicated. Cells were serum-starved for 48 hours, followed by another 48 hours of serum starvation (A and B) or incubation with 10µM H_2O_2 for 8 hours (C and D) to induce apoptosis. Cells were harvested and subjected to flow cytometry to analyse VSMC apoptosis. Data presented here are averaged (mean±S.E.M.) from four independent experiments.

Supplemental Figure 5. Validated/Predicted miR-22 target gene expression profiles in VSMCs treated with TGFβ1.

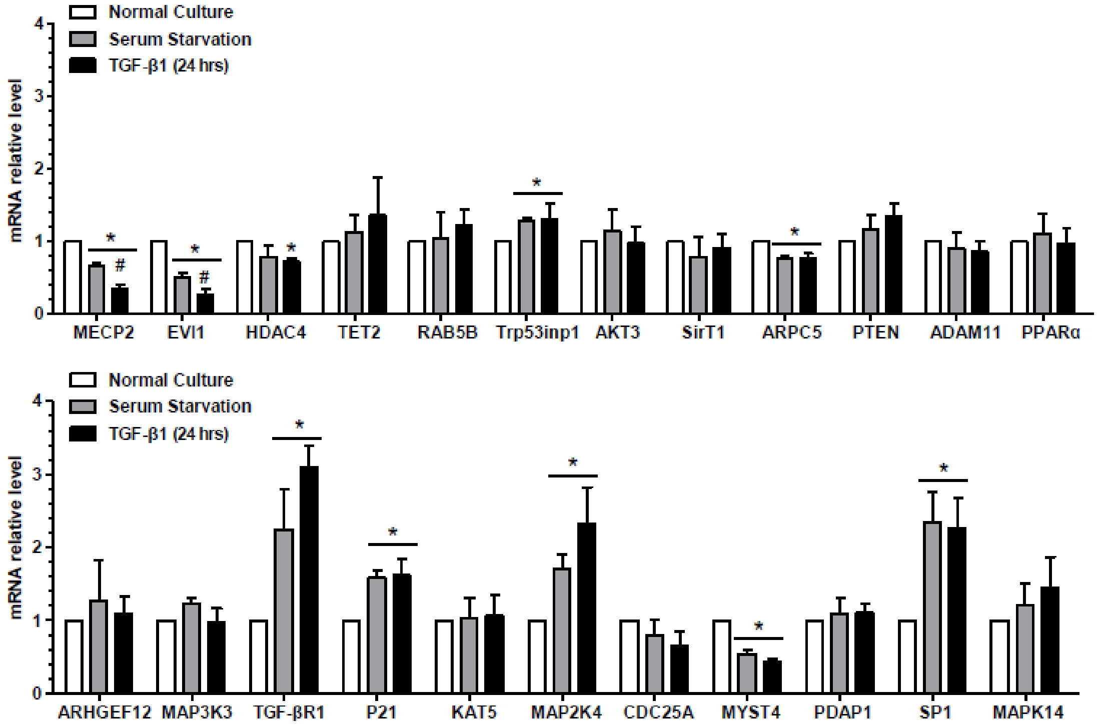

Total RNAs were harvested from VSMCs under normal culture condition (Normal culture), serum starvation for 48 hours (Serum starvation), or serum starvation for 48 hours followed by a further 24 hours of TGF-β1 (5 ng/ml) stimulation (TGF-β1, 24 hrs). RT-qPCR analyses were performed to examine the expression levels of validated/predicted target genes of miR-22. Data presented here are averaged from three independent experiments (n=3). *P<0.05 (versus normal culture), $^{\#}$P<0.05 (versus serum starvation).

Supplemental Figure 6. Role of MECP2, a target gene of miR-22, in VSMC phenotypic modulation.

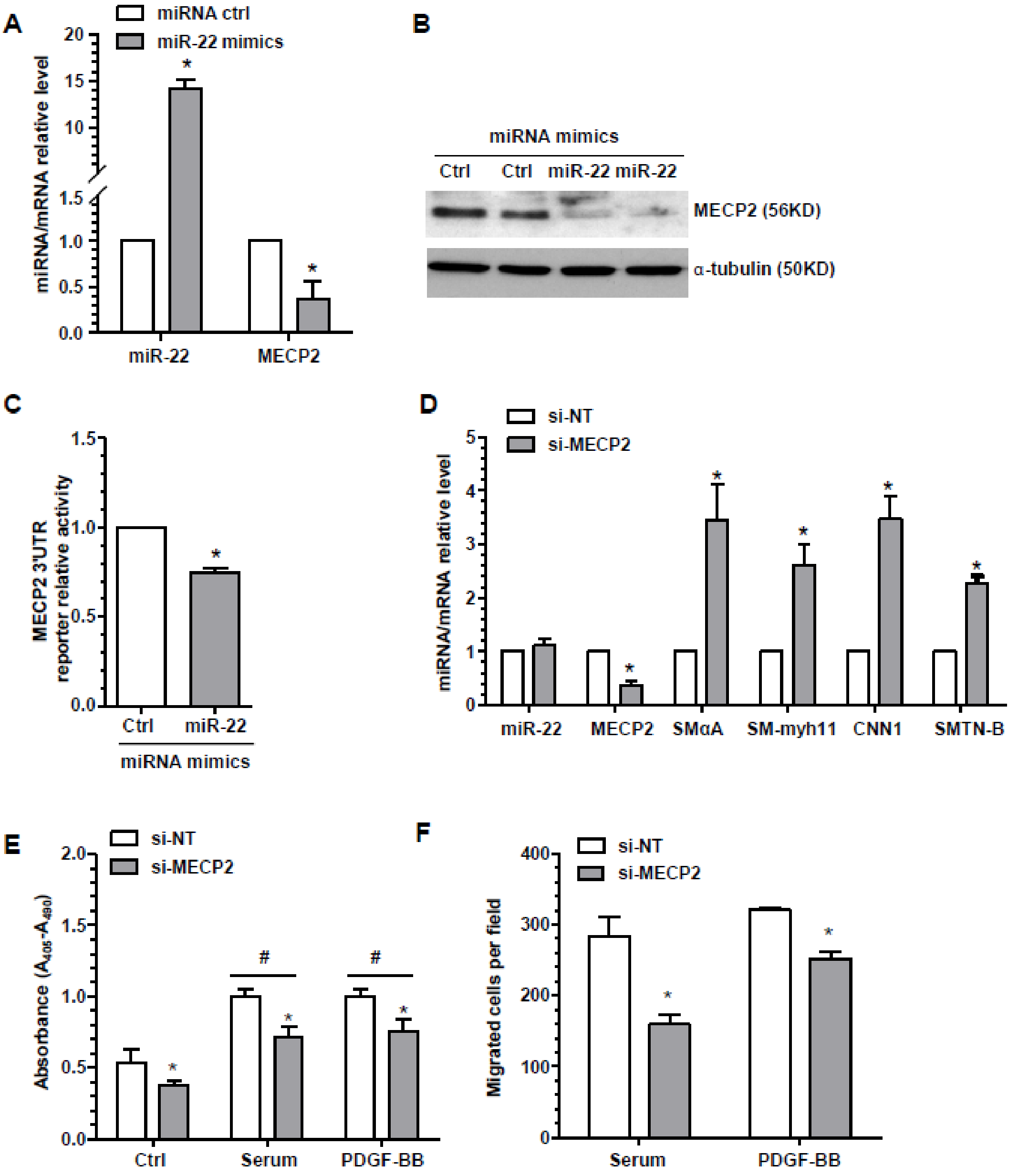

(A-B) miR-22 over-expression represses MECP2 in VSMCs. VSMCs were transfected with miR-22 mimics or control miRNAs (miRNA ctrl), respectively. Total RNAs and proteins were harvested and subjected to RT-qPCR (A) and Western blot (B) analyses, respectively. (C) miR-22 over-expression inhibits MECP2 3'UTR reporter activity. VSMCs were co-transfected with MECP2 3'UTR reporter (pmiR-Luc-MECP2) and miR-22 mimics or control miRNAs (miRNA ctrl), respectively. Cell lysate were harvested and subjected to luciferase activity assays. (D-F) MECP2 inhibition recapitulates the effects of miR-22 over-expression on VSMC specific gene expression, proliferation, and migration. VSMCs transfected with control (non-target siRNA, [si-NT]) or MECP2 specific siRNAs (si-MECP2) were subjected to serum starvation for 48 hours. Cells were harvested for RT-qPCR to measure gene

expression (D). BrdU incorporation (E) and trans-well migration (F) assays were performed to measure the proliferation and migration of transfected and serum-starved cells in response to 20% serum or PDGF-BB stimulation. The concentration of PDGF-BB was 10 mg/mL and 30 mg/mL for BrdU incorporation and trans-well migration assays, respectively. Note: very few or zero migrated cells were observed without cell chemoattractant in trans-well migration assays; thus, control is not shown. Data presented here are averaged from three independent experiments (n=3). *P<0.05 (versus miRNA ctrl or si-NT), $^{\#}$P<0.05 (versus Ctrl).

Supplemental Figure 7. Role of HDAC4 in VSMC phenotypic modulation.

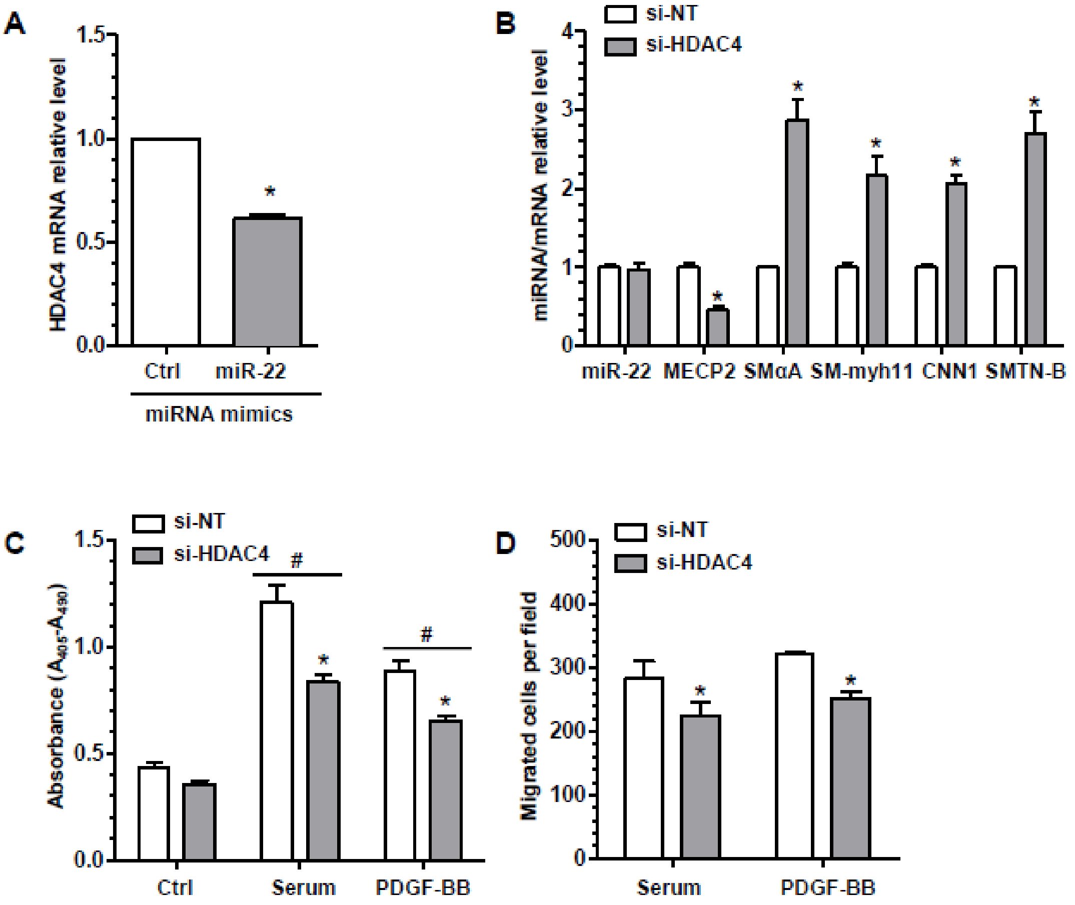

(A) miR-22 over-expression represses HDAC4 in VSMCs. VSMCs were transfected with miR-22 mimics or control miRNAs (miRNA ctrl), respectively. Total RNAs were harvested and subjected to RT-qPCR analyses. (B-D) HDAC4 inhibition recapitulates the effects of miR-22 over-expression on VSMC specific gene expression, proliferation, and migration. VSMCs transfected with control (non-target siRNA, [si-NT]) or HDAC4 siRNAs (si-HDAC4) were subjected to serum starvation for 48 hours. Cells were harvested for RT-qPCR to measure gene expression (B). BrdU incorporation (C) and trans-well migration (D) assays were performed to measure the proliferation and migration of transfected and serum-starved cells in response to 20% serum or PDGF-BB stimulation. The concentration of PDGF-BB was 10 mg/mL and 30 mg/mL for BrdU incorporation and trans-well migration assays, respectively. Note: very few or zero migrated cells were observed without cell chemoattractant in trans-well migration assays; thus, control is not shown. Data presented here are averaged from three independent experiments (n=3). *P<0.05 (versus miRNA ctrl or si-NT), $^{\#}$P<0.05 (versus Ctrl).

Supplemental Figure 8. EVI1 repression is required for miR-22 mediated VSMC phenotypic modulation.

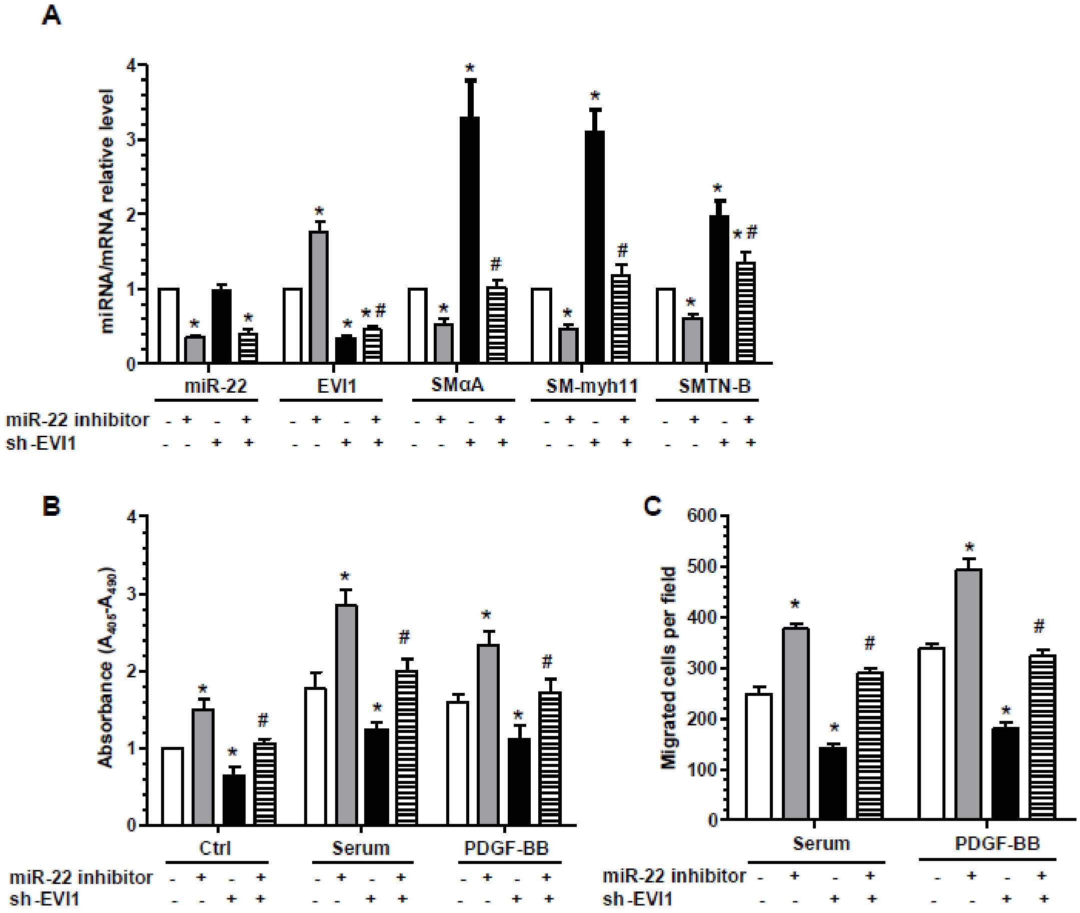

(A) Control (sh-EVI1-) and EVI1 stable knockdown (sh-EVI1+) VSMCs were transfected with miR-22 inhibitor (miR-22 inhibitor+) or control miRNA inhibitor (miR-22 inhibitor-) as indicated. Transfected cells were serum-starved for 48 hours and subjected to RT-qPCR analyses for gene expression. (B&C) Control (sh-EVI1-) and EVI1 stable knockdown (sh-EVI1+) VSMCs were transfected with miR-22 inhibitor (miR-22 inhibitor+) or control miRNA inhibitor (miR-22 inhibitor-). Subsequently, transfected cells were subjected to serum starvation for 48 hours, followed by BrdU incorporation assays (B) or trans-well migration (C) in response to Ctrl (0% serum), 20% serum or PDGF-BB stimulation. The concentration of PDGF-BB was 10 mg/mL and 30 mg/mL for BrdU incorporation and trans-well migration assays, respectively. Note: very few or zero migrated cells were observed without cell chemoattractant in trans-well migration assays; thus, control is not shown. Data presented here are averaged (mean±S.E.M.) from three independent experiments. *P<0.05 (versus miR-22 inhibitor-, sh-EVI1-); #P<0.05 (miR-22 inhibitor+, sh-EVI1+ versus miR-22 inhibitor+, sh-EVI1-).

Supplemental Figure 9. Expression profiles of miR-22 and target genes in the healthy and diseased human arteries.

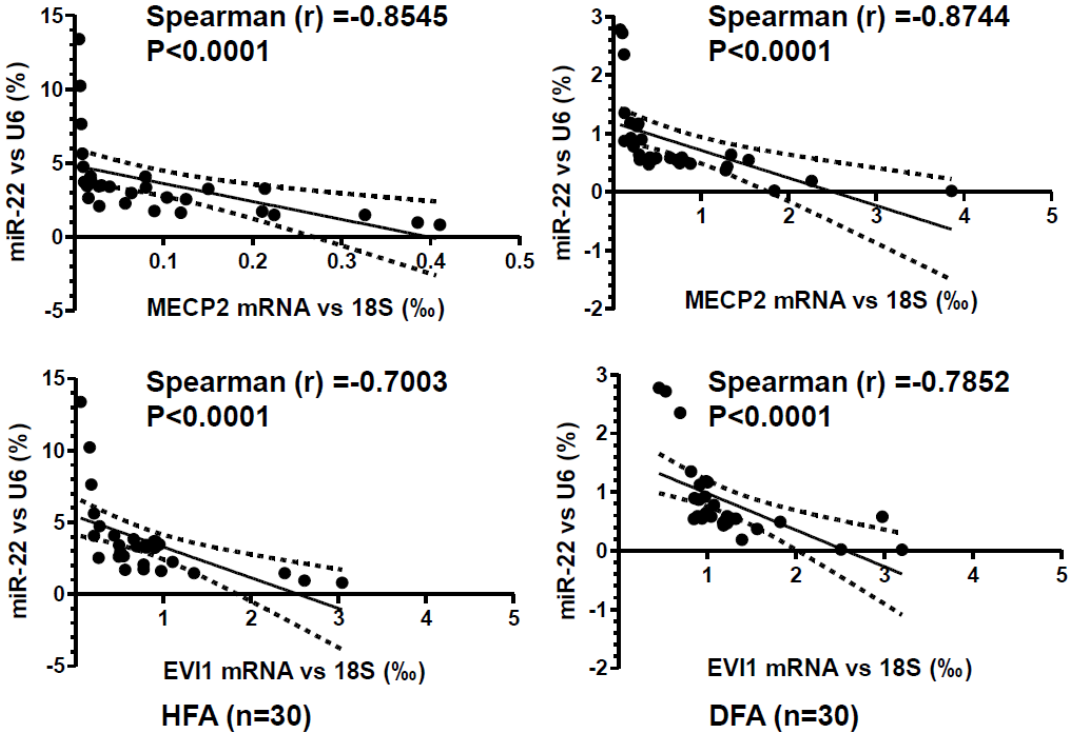

Healthy femoral artery specimens (HFA, n=30) from patients without peripheral arterial diseases and diseased femoral artery specimens (DFA, n=30) from patients with peripheral arterial diseases were collected and subjected to RT-qPCR analyses. Spearman's rank correlation analyses were carried out to characterize the relationships between the gene expression levels of MECP2/ EVI1 and miR-22 in HFA and DFA specimens. Y-axis represents expression level of miR-22 (relative to U6, %); X-axis represents the expression level of its target genes (MECP2 and EVI1) (relative to 18S, ‰). The solid line indicates the fitted linear regression line; the dotted line indicates 95% CI level. R is Pearson's correlation coefficient between the expression levels of MECP2/EVI1 and miR-22. A value closer to -1 indicates a stronger negative correlation, and a value closer to 1 indicates a stronger positive correlation. P is the P-value indicating the significant level of correlation.
